# Spatial distribution of live gut microbiota and bile acid metabolism in various parts of human large intestine

**DOI:** 10.1038/s41598-022-07594-6

**Published:** 2022-03-04

**Authors:** Daisuke Chinda, Toshihiko Takada, Tatsuya Mikami, Kensuke Shimizu, Kosuke Oana, Tetsu Arai, Kazuki Akitaya, Hirotake Sakuraba, Miyuki Katto, Yusuke Nagara, Hiroshi Makino, Daichi Fujii, Kenji Oishi, Shinsaku Fukuda

**Affiliations:** 1 Department of Gastroenterology and Hematology, Hirosaki University Graduate School of Medicine, 5 Zaifu-cho, Hirosaki, Aomori 036-8562 Japan; 2 Division of Endoscopy, Hirosaki University Hospital, Hirosaki, Aomori Japan; 3 Basic Research Department, Yakult Central Institute, Tokyo, Japan; 4 Innovation Center for Health Promotion, Hirosaki University Graduate School of Medicine, Hirosaki, Aomori Japan; 5 Food Research Department, Yakult Central Institute, Tokyo, Japan; 6 Microbiological Research Department, Yakult Central Institute, Tokyo, Japan

**Keywords:** Gastroenterology, Medical research

## Abstract

Gut microbiomics is based on analysis of both live and dead cells in the stool. However, to understand the ecology of gut microbiota and their symbiotic relationships with hosts, spatial distribution of live bacteria must be examined. Here, we analyzed the live composition of luminal microbiota (LM) and mucosa-associated microbiota (MAM) in the ascending and descending colons and the rectums of 10 healthy adults and compared it with the total composition. The abundance of *Lachnospiraceae* in live LM decreased along the gut length and was significantly lower than that in total LM. Contrastingly, the abundance of *Bacteroidaceae* and *Bifidobacteriaceae* in live LM was higher than that in total LM, suggesting differences in death rate during gut migration. Live *Enterobacteriaceae* levels in MAM were significantly higher in rectum than in the ascending and descending colons and in LM. High-performance liquid chromatographic analysis of luminal bile acids revealed that 7α-dehydroxylation occurred towards the rectum. In live LM where a bile acid-inducible gene could be detected*,* 7α-dehydroxylation rates were higher than those in the group without the gene. Overall, we showed differences in live bacteria composition among three gut sites and between LM and MAM, highlighting the importance of understanding their spatial distribution.

## Introduction

There are more than 100 trillion bacteria from around 1,000 species in the human colon, forming an extremely complex microbial ecosystem^1^. This ecosystem, referred to as the gut microbiota, plays important nutritional, immunological, and clinical roles^2–4^. Therefore, the complete understanding of the gut microbiota is very important to develop strategies that support the maintenance of lifelong health in humans.

Recently, the use of comprehensive analytical technologies, such as next-generation sequencing, has become the mainstream approach to analyzing complex gut microbiota. More often than not, stool samples are used in these investigations because they are easy to handle and can be collected using non-invasive strategies. However, the analysis of microbiota in stool samples does not reflect bacterial assemblages present in different parts of the gut. For example, in humans, the left-sided colon is distinct from the right-sided colon, not only in terms of physiological functions such as nerve and blood vessel control and gut motility, but also in terms of cell composition^5–9^. Therefore, it is unsurprising that they also differ in terms of the ecology of intestinal bacteria. Ever since Eckburg et al.^10^ reported their colon site-specific analysis of microbiota via 16S rRNA, many researchers have analyzed the composition of the microbiota collected from various parts of the gut (both horizontal and vertical distribution^11–15^. These studies showed that the gut microbiota in healthy people varies more among individuals than among parts of the gut. However, in these studies, no distinction was made between live and dead bacteria. Ben-Amor et al. (2005) analyzed the microbiota in stool samples after controlling for bacterial viability using the permeability of the bacterial cell membrane as an indicator and reported that only ~ 50% of the bacteria were alive^16^. To date, no study has reported the investigation of the microbiota gut distribution by focusing on live bacteria alone.

Propidium monoazide (PMA) specifically binds to the DNA of dead cells, as the cell membrane in live cells is impermeable to PMA; therefore, the PMA-bound DNA is not amplified by PCR, and only the DNA from live cells is detected. Using 16S rRNA meta-analysis of PMA-treated and untreated samples, Fu et al. (2018) reported site differences (foregut vs. hindgut) in the proportion and composition of microbiome of total versus live bacteria in the gut of rex rabbits^17^. Moreover, Tian et al. (2017) reported a correlation between the results of an approach involving a combination of PMA and MiSeq (PMA-MiSeq) and ATP activity (an indicator of viability) of bacteria inhabiting sludge, suggesting that PMA-MiSeq is useful for analyzing live bacteria^18^.

Apart from the bacteria themselves, bacterial metabolism-derived products, such as 7α-dehydroxylated bile acids, may also affect host health. Dehydroxylation of the 7α-hydroxyl group of bile acids produces deoxycholic and lithocholic acids, which have potent cytotoxic activity and have been suggested to promote colon cancer in many experimental animal models^19^. However, no study has identified the exact part of the gut in which individual bacteria and metabolites were produced. These hypotheses need to be explored to better understand the symbiotic relationship between intestinal bacteria and the host. Particularly, a focus on metabolically active, live bacteria is needed.

Here, we sampled the contents of the gut (including stool) and mucosal scraping of the ascending colon, descending colon, and rectum of healthy adult participants using colonoscopy for the analysis of luminal microbiota (LM) and mucosa-associated microbiota (MAM), respectively. We then compared the live and total compositions of LM and MAM using PMA-MiSeq to clarify whether there are horizontal and/or vertical spatial distribution differences in gut microbiota composition. In addition, we analyzed bile acid composition in the contents of each gut sample (including stool) to identify the areas where 7α-dehydroxylation occurred. Finally, we compared this with the spatial distribution of the respective live LM, aiming to understand the relationship between live bacteria possessing bile acid-inducible (*bai*) genes and 7α-dehydroxylation.

## Results

### Bacterial composition in LM (including stool) and MAM from each part of the gut

**Figure 1:**
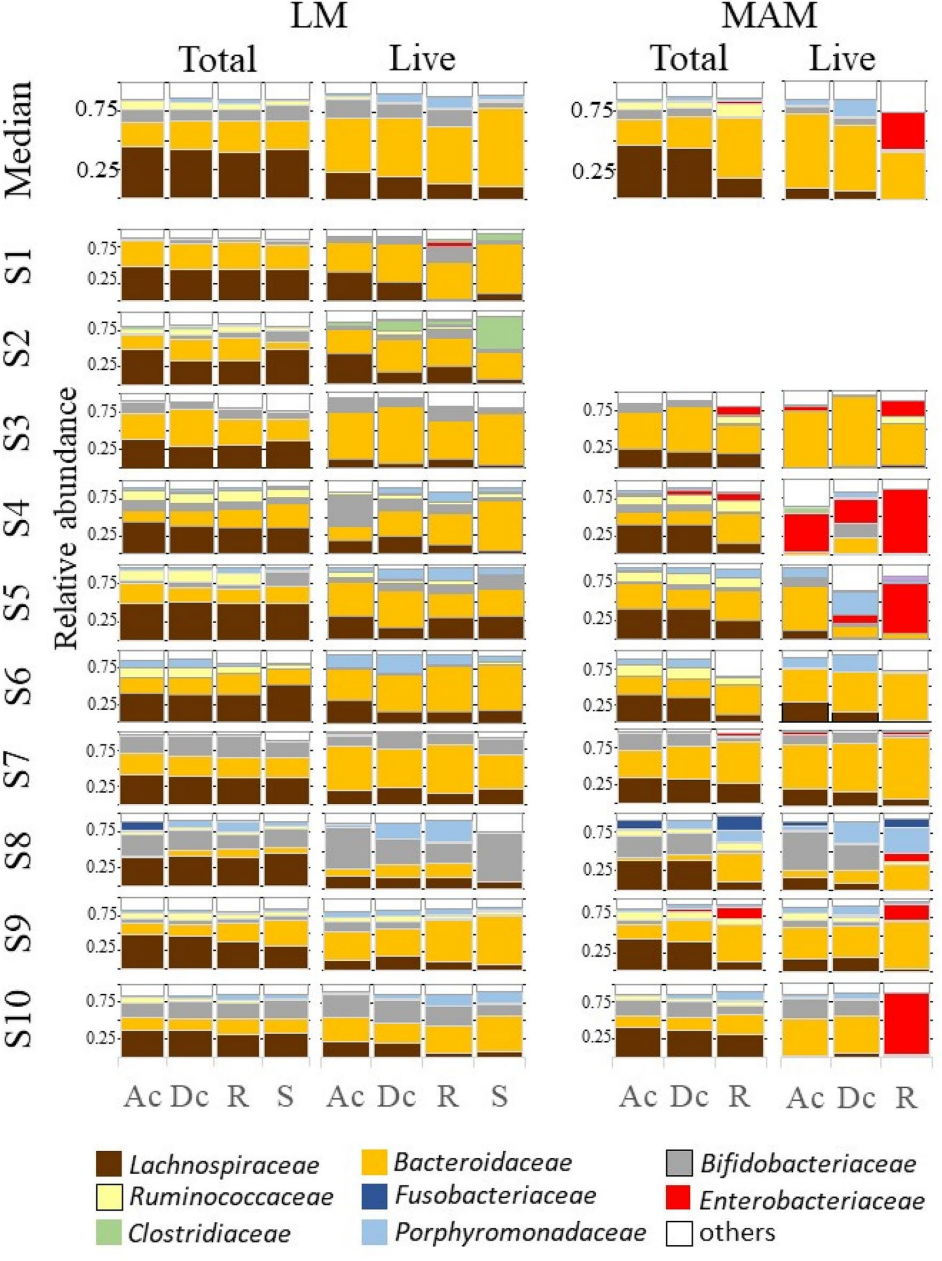
Relative abundance of total and live bacterial families within the luminal microbiota (LM) and mucosa-associated microbiota (MAM) from each part of the gut. Bacterial family distribution of the operational taxonomic units (OTUs). The relative abundance of total bacteria and live bacteria within LM from 10 subjects were analyzed. The relative abundance of total bacteria and live bacteria within MAM from only 8 subjects are shown; due to the small amount of DNA obtained from mucosal scraping samples of subjects 1 and 2, their respective results were not included. Eight major contributing families are displayed in different colors and other minor contributing families are grouped and displayed in white. LM: luminal microbiota; MAM: mucosa-associated microbiota; OTU: operational taxonomic unit; Ac: ascending colon; Dc: descending colon; R: rectum; S: stool.

A total of 140 gut content (including stool)/mucosal scraping samples collected from different parts of the gut were used for amplicon sequencing analysis. We obtained a total of 2,858,432 reads (minimum, 5,282 reads; maximum, 40,600 reads; mean, 20,417 reads), and identified a total of 1,995 Feature IDs. Of note, there was no difference in the mean number of reads detected between total and live bacteria (*P* = 0.548): 20,029 ± 8,012 and 20,806 ± 6,958 reads, respectively. The relative abundance of individual total bacteria and live bacteria within LM and MAM from each part of the gut, as well as medians, are shown in Fig. 1. Of note, due to the small amount of DNA obtained from mucosal scraping samples of subjects 1 and 2, their respective results were not considered.

(1) LM composition.

**Figure 2:**
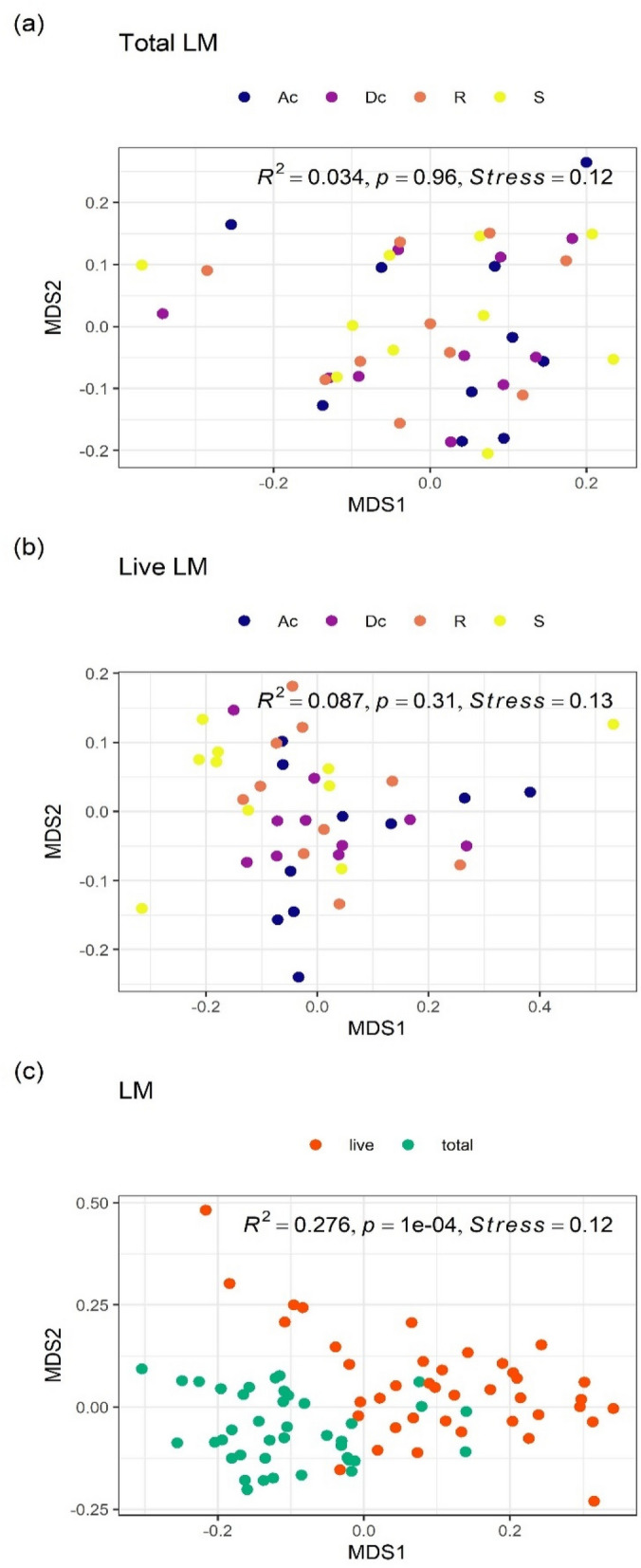
NMDS ordinations and PERMANOVA analyses of LM composition. (**a**) Overall comparison of total LM composition among three gut sites and stool, (**b**) overall comparison of live LM composition among three gut sites and stool, (**c**) overall comparison between total and live LM composition. All ordinations and PERMANOVA analyses were performed using Bray–Curtis distance matrix based on relative abundance of taxa at the family level. LM: luminal microbiota; NMDS: Non-metric multidimensional scaling; Ac: ascending colon; Dc: descending colon; R: rectum; S: stool.

Non-metric multidimensional scaling (NMDS) ordination and PERMANOVA analysis showed no significant differences (R^2^ = 0.034, *P* = 0.96, Fig. 2a) in dissimilarity of total LM composition among the three gut sites (ascending colon, descending colon, and rectum) and stool. There was also no difference in the abundance of bacterial groups with a mean relative abundance of 1% or more among all three gut sites and stool, indicating that the microbiota composition was similar. The predominant bacterial groups in total LM composition at all three gut sites and in stool were *Lachnospiraceae*, *Bacteroidaceae*, and *Bifidobacteriaceae*, accounting for median relative abundances of about 40%, 25%, and 10%, respectively (Fig. 1).

The dissimilarity in the live LM composition did not differ significantly among all three gut sites and stool (PERMANOVA: R^2^ = 0.087, *P* = 0.31, Fig. 2b). On the other hand, the abundance of a few bacterial groups differed among all three gut sites and stool. For example, the abundance of *Lachnospiraceae* gradually decreased in order of ascending colon, descending colon, rectum, and stool (Fig. 1 and Table 1).

**Table 1:** Differentially abundant taxa at family level among four gut sites (ascending colon, descending colon, rectum, and stool) in the live LM.

Family	Relative abundance (%)	Padj	Gut sites compared	log2FC
*Lachnospiraceae*	15.8	0.0012	Rectum vs Stool	0.3
			Descending colon vs Stool	0.7
			Ascending colon vs Stool	1.0
*Veillonellaceae*	2.1	0.011	Rectum vs Stool	2.5
			Descending colon vs Stool	2.2
			Ascending colon vs Stool	2.3

The live LM samples (n = 40; ten subjects over four gut sites) were used in DESeq2 analysis with full model of ~ subject + gut_sites and reduced model of ~ subject.

Criteria for inclusion: Relative abundance > 1% and padj < 0.01.

Relative abundance: mean on relative abundance of normalized counts for subset samples.

padj: the Benjamini–Hochberg adjusted *P* value.

log2FC: log2(fold change).

Gut sites compared: e.g., “Rectum vs Stool” means that log2FC means log2(Rectum/Stool).

*LM* luminal microbiota.

The dissimilarity of total LM composition differed significantly from that of the live LM composition (PERMANOVA: R^2^ = 0.276, *P* = 1e−4, Fig. 2c). For each bacterial group, the abundance of *Bacteroidaceae* and *Bifidobacteriaceae* in the live LM was significantly higher than that in total LM, whereas the abundance of *Lachnospiraceae* was significantly lower in the live LM (Table 2). The same trend was observed for bacterial groups classified at the 97% operational taxonomic unit (OTU) level (Table 3).

**Table 2:** Differentially abundant taxa at family level between total and live bacteria in the LM.

Family	Relative abundance (%)	Padj	log2FC
*Bacteroidaceae*	34.9	2.0e−18	1.3
*Lachnospiraceae*	25.7	2.3e−15	− 0.9
*Bifidobacteriaceae*	16.0	0.0019	0.7
*Porphyromonadaceae*	5.5	2.9e− 06	1.5
*Coriobacteriaceae*	4.5	4.0e−05	0.8
*Ruminococcaceae*	3.8	0.00026	− 0.7
*o__Clostridiales;*	1.4	0.011	1.6
*Erysipelotrichaceae*	1.2	5.1e−06	− 1.3
*Streptococcaceae*	1.0	6.9e−07	− 2.8

The LM samples (n = 80; ten subjects, four gut sites over total/live) were used in DESeq2 analysis with full model of ~ subject + gut_sites + live and reduced model of ~ subject + gut_sites, where "live" means live or total bacteria.

Criteria for inclusion: Relative abundance > 1% and padj < 0.01.

Relative abundance: mean on relative abundance of normalized counts for subset samples.

padj: the Benjamini–Hochberg adjusted *P* value.

log2FC: log2(live/total).

*LM* luminal microbiota.

**Table 3:** Differentially abundant OTUs in the family *Bacteroidaceae/ Lachnospiraceae/ Bifidobacteriaceae* between total and live bacteria in the LM.

Family	Taxonomic annotation	Similarity (%)	Relative abundance (%)	padj	log2FC
*Bacteroidaceae*	*Bacteroides vulgatus*	100	6.21	0.0014	1.0
	*Bacteroides dorei*	100	5.08	0.00058	1.5
	*Bacteroides uniformis*	100	4.61	1.3e−06	1.8
	*Bacteroides ovatus*	99.4	2.54	4.2e−05	1.9
	*Bacteroides xylanisolvens*	100	1.67	4.2e−05	1.5
	*Bacteroides ovatus*	99	0.75	0.0057	1.4
	*Bacteroides thetaiotaomicron*	99.1	0.43	0.00040	1.6
*Lachnospiraceae*	*Anaerostipes hadrus*	100	2.30	0.0024	− 1.8
	*Fusicatenibacter saccharivorans*	99.7	1.70	4.2e−05	− 1.6
	*[Ruminococcus] gnavus*	100	1.13	0.00024	− 1.3
	*Anaerostipes hadrus*	99.7	0.72	0.00038	− 1.9
	*Fusicatenibacter saccharivorans*	96.3	0.65	0.0065	− 1.6
	*[Clostridium] indolis*	94.5	0.19	0.0024	− 2.5
*Bifidobacteriaceae*	*Bifidobacterium longum infantis*	99.4	3.49	0.0097	0.9

The LM samples (n = 80; ten subjects, total/live over four gut sites) were used in DESeq2 analysis with full model of ~ subject + gut_sites + live and reduced model of ~ subject + gut_sites, where "live" means live or total bacteria.

Criteria for inclusion: padj < 0.01.

Relative abundance; mean on relative abundance of normalized counts for subset samples.

padj: the Benjamini–Hochberg adjusted *P* value.

log2FC: log2(live/total).

*LM* luminal microbiota, *OTU* operational taxonomic unit.

(2) MAM composition.

**Figure 3:**
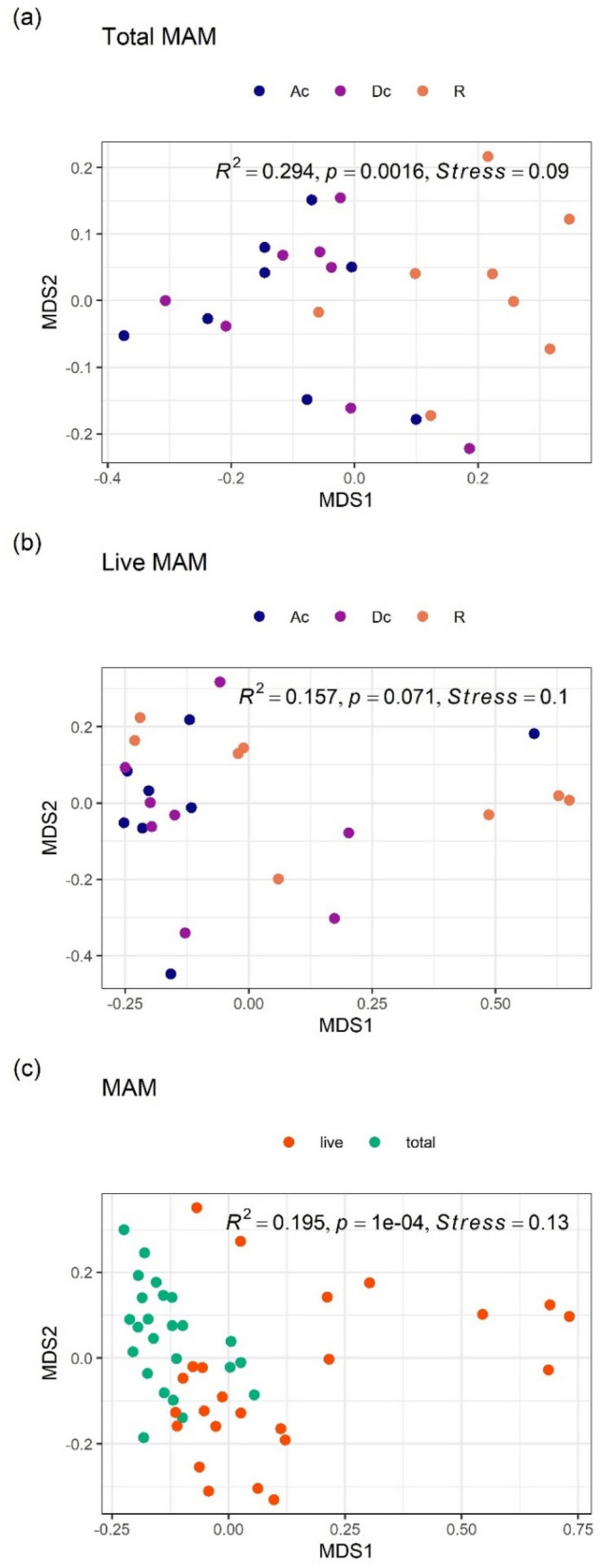
NMDS ordinations and PERMANOVA analyses of MAM composition. (**a**) Overall comparison of total MAM composition among three gut sites, (**b**) overall comparison of live MAM composition among three gut sites, (**c**) overall comparison between total and live MAM composition. All ordinations and PERMANOVA analyses were performed using Bray–Curtis distance matrix based on relative abundance of taxa at the family level. NMDS: Non-metric multidimensional scaling; MAM: mucosa-associated microbiota; Ac: ascending colon; Dc: descending colon; R: rectum.

The dissimilarity of total MAM composition differed significantly among the three gut sites (PERMANOVA: R^2^ = 0.294, *P* = 0.0016, Fig. 3a). Pairwise PERMANOVA analysis revealed that the dissimilarity of total MAM composition differed significantly between the rectum and both the ascending colon (R^2^ = 0.278, adjusted *p*-value = 0.009) and the descending colon (R^2^ = 0.349, adjusted *p*-value = 0.003). On the other hand, no significant difference was detected between the ascending and descending colons (R^2^ = 0.019, adjusted *p*-value = 0.826). In other words, the total MAM composition was similar from the ascending colon to the descending colon, but the composition changed markedly in the rectum. Focusing on each bacterial group (Table 4), the abundance of *Lachnospiraceae* was significantly higher in both the ascending and descending colons than in the rectum. In contrast, the abundance of *Bacteroidaceae* and *Enterobacteriaceae* was significantly higher in the rectum than in both the ascending and descending colons.

**Table 4:** Differentially abundant taxa at family level among three gut sites (ascending colon, descending colon, and rectum) in the total MAM.

Family	Relative abundance (%)	padj	Gut sites compared	log2FC
*Bacteroidaceae*	30.5	0.00055	Descending colon vs Rectum	− 1.0
			Ascending colon vs Rectum	− 1.1
*Lachnospiraceae*	30.1	0.00034	Descending colon vs Rectum	0.6
			Ascending colon vs Rectum	0.9
*Ruminococcaceae*	8.4	0.034	Descending colon vs Rectum	− 1.0
			Ascending colon vs Rectum	− 0.8
*Enterobacteriaceae*	2.6	2.7e−05	Descending colon vs Rectum	− 3.1
			Ascending colon vs Rectum	− 3.1
*Erysipelotrichaceae*	2.0	0.00049	Descending colon vs Rectum	− 1.8
			Ascending colon vs Rectum	− 2.0
*Fusobacteriaceae*	1.9	0.00071	Descending colon vs Rectum	− 2.6
			Ascending colon vs Rectum	− 2.9
*Streptococcaceae*	1.1	0.047	Descending colon vs Rectum	0.8
			Ascending colon vs Rectum	1.7

The total MAM samples (n = 24, eight subjects over three gut sites) were used in DESeq2 analysis with full model of ~ subject + gut_sites and reduced model of ~ subject.

Criteria for inclusion: Relative abundance > 1% and padj < 0.01.

Relative abundance; mean on relative abundance of normalized counts for subset samples.

padj; the Benjamini–Hochberg adjusted *P* value.

log2FC; log2(fold change).

Gut sites compared; e.g., “Descending colon vs Rectum” means that log2FC means log2(Descending colon/rectum).

*MAM* mucosa‒associated microbiota.

NMDS ordination and PERMANOVA analysis of the dissimilarity in the live MAM composition showed no significance among all three gut sites (R^2^ = 0.157, *P* = 0.071, Fig. 3b). However, the abundance of *Enterobacteriaceae* was significantly higher in the rectum than in both the ascending and descending colons (Table 5).

**Table 5:** Differentially abundant taxa at family level among three gut sites (ascending colon, descending colon, rectum) in the live MAM.

Family	Relative abundance (%)	padj	Groups compared	log2FC
*Enterobacteriaceae*	25.1	0.00055	Descending colon vs Rectum	− 4.2
			Ascending colon vs Rectum	− 3.7

The live MAM samples (n = 24, eight subjects over three gut sites) were used in DESeq2 analysis with full model of ~ subject + gut_sites and reduced model of ~ subject.

Criteria for inclusion: Relative abundance > 1% and padj < 0.01.

Relative abundance; mean on relative abundance of normalized counts for subset samples.

padj: the Benjamini–Hochberg adjusted *P* value.

log2FC: log2(fold change).

Gut sites compared: e.g., “Descending colon vs Rectum” means that log2FC means log2(Descending colon/rectum).

*MAM* mucosa‒associated microbiota, *LM* luminal microbiota.

The dissimilarity between total MAM composition and the live MAM composition was significant (PERMANOVA: R^2^ = 0.195, *P* = 1e−4, Fig. 3c). Significant differences were detected in many bacterial groups (Table 6). For example, the abundances of live *Bacteroidaceae* and *Enterobacteriaceae* were higher in MAM, whereas the abundance of live *Lachnospiraceae* was lower in MAM.

**Table 6:** Differentially abundant taxa at family level between total and live bacteria in the MAM.

Family	Relative abundance (%)	Padj	log2FC
*Bacteroidaceae*	33.2	0.022	0.7
*Enterobacteriaceae*	16.9	2.8e−06	3.0
*Lachnospiraceae*	15.4	1.2e−06	− 2.1
*Porphyromonadaceae*	6.3	0.0012	1.3
*Ruminococcaceae*	3.9	6.8e−06	− 2.1
*Veillonellaceae*	2.6	1.2e−06	1.8
*Coriobacteriaceae*	1.9	0.022	− 1.0
*Erysipelotrichaceae*	1.5	0.0042	− 1.7
*Turicibacteraceae*	1.2	0.0045	5.1

The MAM samples (n = 48; eight subjects, three gut sites over total/live) were used in DESeq2 analysis with full model of ~ subject + gut_sites + live and reduced model of ~ subject + gut_sites, where "live" means live or total bacteria.

Criteria for inclusion: Relative abundance > 1% and padj < 0.01.

Relative abundance: mean on relative abundance of normalized counts for subset samples; padj: the Benjamini–Hochberg adjusted *P* value.

log2FC: log2(live/total).

*MAM* mucosa‒associated microbiota.

(3) Comparison of LM and MAM compositions.

**Figure 4:**
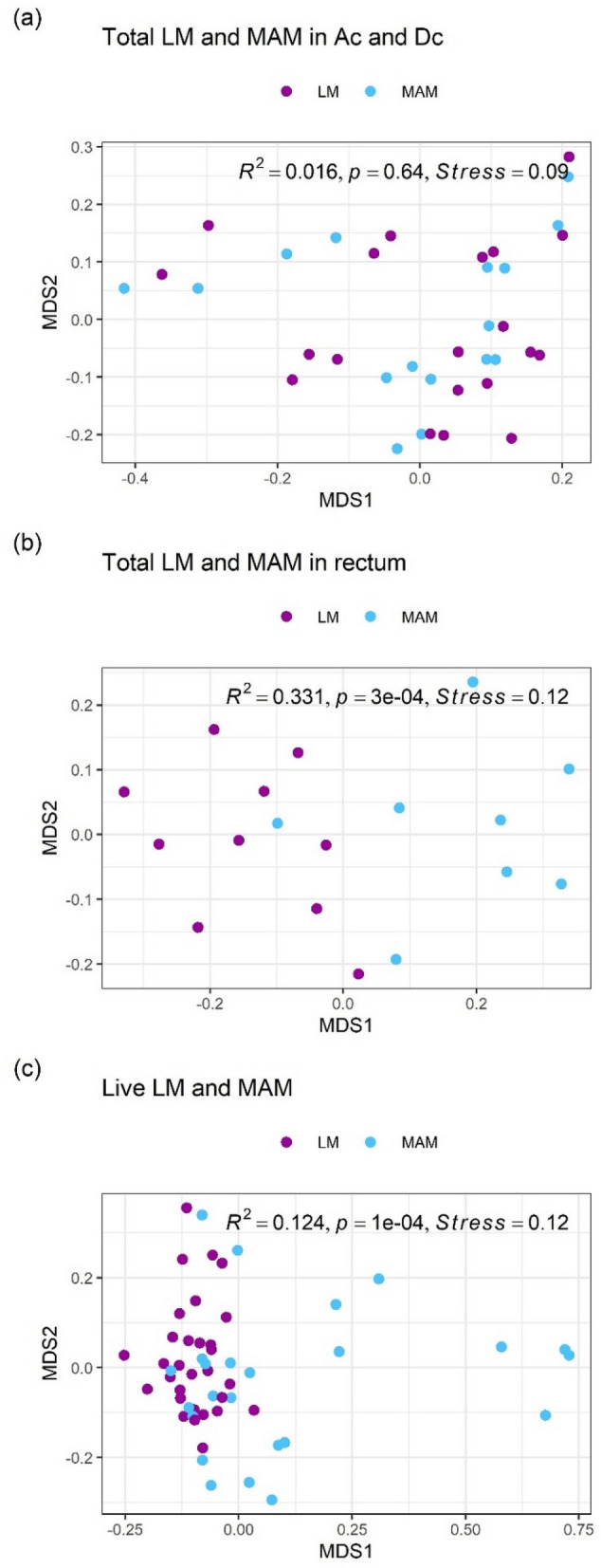
NMDS ordinations and PERMANOVA analyses of LM and MAM composition. (**a**) Overall comparison between total LM and MAM composition in both ascending and descending colons, (**b**) overall comparison between total LM and MAM composition in the rectum, (**c**) overall comparison between live LM and MAM composition in three gut sites. All ordinations and PERMANOVA analyses were performed using Bray–Curtis distance matrix based on relative abundance of taxa at the family level. NMDS: Non-metric multidimensional scaling; MAM: mucosa-associated microbiota; LM: luminal microbiota.

Since the total MAM composition differed significantly between the colon (both ascending and descending) and rectum, as mentioned above, the composition of LM and MAM was compared between these two parts. The dissimilarity in total microbiota composition between LM and MAM in both ascending and descending colons was not significant (PERMANOVA: R^2^ = 0.016, *P* = 0.64, Fig. 4a); for each bacterial group with a mean relative abundance of 1% or more, abundances did not differ significantly between LM and MAM, suggesting that the total LM composition in both the ascending and descending colons was similar to that of MAM. On the other hand, the dissimilarity in total microbiota composition in the rectum differed significantly between LM and MAM (PERMANOVA: R^2^ = 0.331, *P* = 3e−4, Fig. 4b). Focusing on each bacterial group, the abundances of *Bacteroidaceae* and *Enterobacteriaceae* were significantly higher in MAM than in LM, and the abundances of *Lachnospiraceae* and *Bifidobacteriaceae* were significantly lower in MAM (Table 7).

**Table 7:** Differentially abundant taxa at Family level in total bacteria between LM and MAM in rectum.

Family	Relative abundance (%)	Padj	log2FC
*Bacteroidaceae*	33.9	0.00014	1.2
*Lachnospiraceae*	26.4	0.0061	− 0.6
*Bifidobacteriaceae*	7.9	0.0030	− 1.5
*Ruminococcaceae*	7.8	0.00083	1.3
*Erysipelotrichaceae*	3.2	0.011	1.4
*Enterobacteriaceae*	3.0	2.1e−09	4.2
*Fusobacteriaceae*	1.7	0.00022	3.0

The total bacteria in rectum samples (n = 18; ten subjects in LM and eight subjects in MAM) were used in DESeq2 analysis with full model of ~ subject + MAM and reduced model of ~ subject, where "MAM" means MAM or LM.

Criteria for inclusion: Relative abundance > 1% and padj < 0.01.

Relative abundance: mean on relative abundance of normalized counts for subset samples; padj: the Benjamini–Hochberg adjusted *P* value.

log2FC: log2(MAM/LM).

*MAM* mucosa‒associated microbiota, *LM* luminal microbiota.

Because the live MAM composition did not differ significantly among all three gut sites (ascending colon, descending colon, and rectum), we compared the live LM composition with the live MAM composition among gut sites taken together; the dissimilarity between the live LM composition and the live MAM composition was significant (PERMANOVA: R^2^ = 0.124, *P* = 1e− 4, Fig. 4c). Focusing on each bacterial group (Table 8), the abundance of live *Enterobacteriaceae* was particularly high in MAM (log2FC value = 4.8). In contrast, the abundances of live *Bifidobacteriaceae* and *Lachnospiraceae* were lower in MAM.

**Table 8:** Differentially abundant taxa at Family level in live bacteria between LM and MAM.

Family	Relative abundance (%)	Padj	log2FC
*Enterobacteriaceae*	13.9	1.9e−08	4.8
*Lachnospiraceae*	11.7	0.0024	− 1.3
*Bifidobacteriaceae*	11.6	0.047	− 1.3
*Veillonellaceae*	3.0	0.012	1.2
*Coriobacteriaceae*	2.4	0.0057	− 1.2

The live bacteria samples excluding stool samples (n = 54; ten subjects over three sites in the LM and eight subjects over three sites in the MAM) were used in DESeq2 analysis with full model of ~ subject + gut_sites + MAM and reduced model of ~ subject + gut_sites, where "MAM" means MAM or LM.

Criteria for inclusion: Relative abundance > 1% and padj < 0.01.

Relative abundance: mean on relative abundance of normalized counts for subset samples.

Padj: the Benjamini–Hochberg adjusted *P* value; log2FC: log2(MAM/LM).

*MAM* mucosa‒associated microbiota, *LM* luminal microbiota.

#### α-Diversity of the microbiota

**Figure 5:**
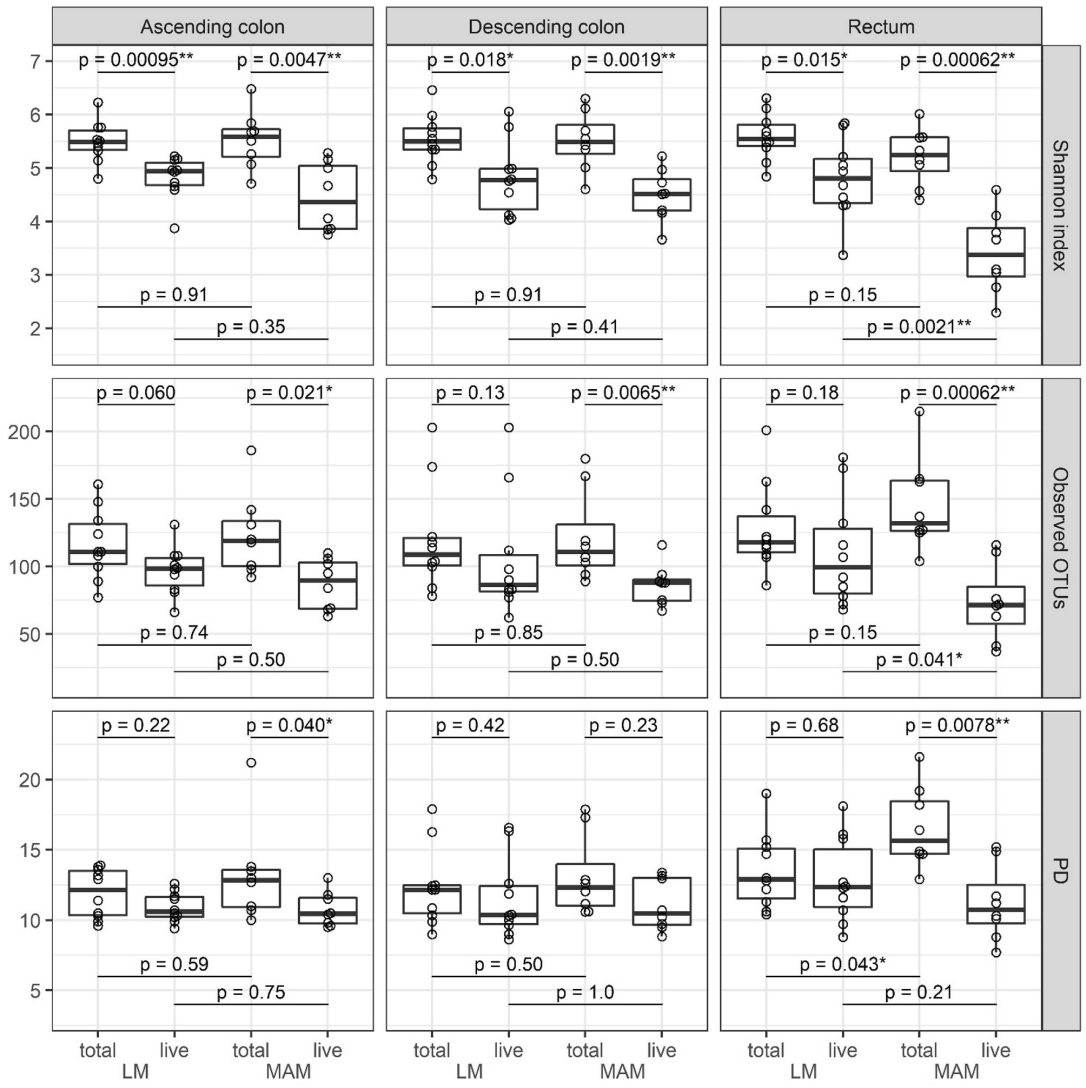
Comparison of the α-diversity of total and live bacteria in LM and MAM from each part of the gut. Top row: results of Shannon index; center: observed OTUs; and bottom: PD for the ascending colon, descending colon, and rectum as labeled. Statistical analysis was performed using the Wilcoxon's rank-sum test: *P < 0.05, **P < 0.01. LM: luminal microbiota; MAM: mucosa-associated microbiota; OTU: operational taxonomic unit; MAM: mucosa-associated microbiota; LM: luminal microbiota; total: total bacteria; live: live bacteria.

In terms of the Shannon index, live bacteria showed significantly lower diversity than total bacteria in both LM and MAM from all parts of the gut (Fig. 5). In terms of observed operational taxonomic units (observed OTUs) and phylogenetic diversity (PD), from all parts of the gut, no significant differences between live and total bacteria were observed in LM, whereas live bacteria showed significantly lower diversity than total bacteria in MAM, except for PD in the descending colon. Furthermore, in the context of LM versus MAM, no significant differences were observed in the ascending and descending colon, but a significant difference was observed in live bacteria for observed OTUs and Shannon index and total bacteria for PD in the rectum.

#### Bile acid composition in contents from various part of the gut and stool

**Figure 6:**
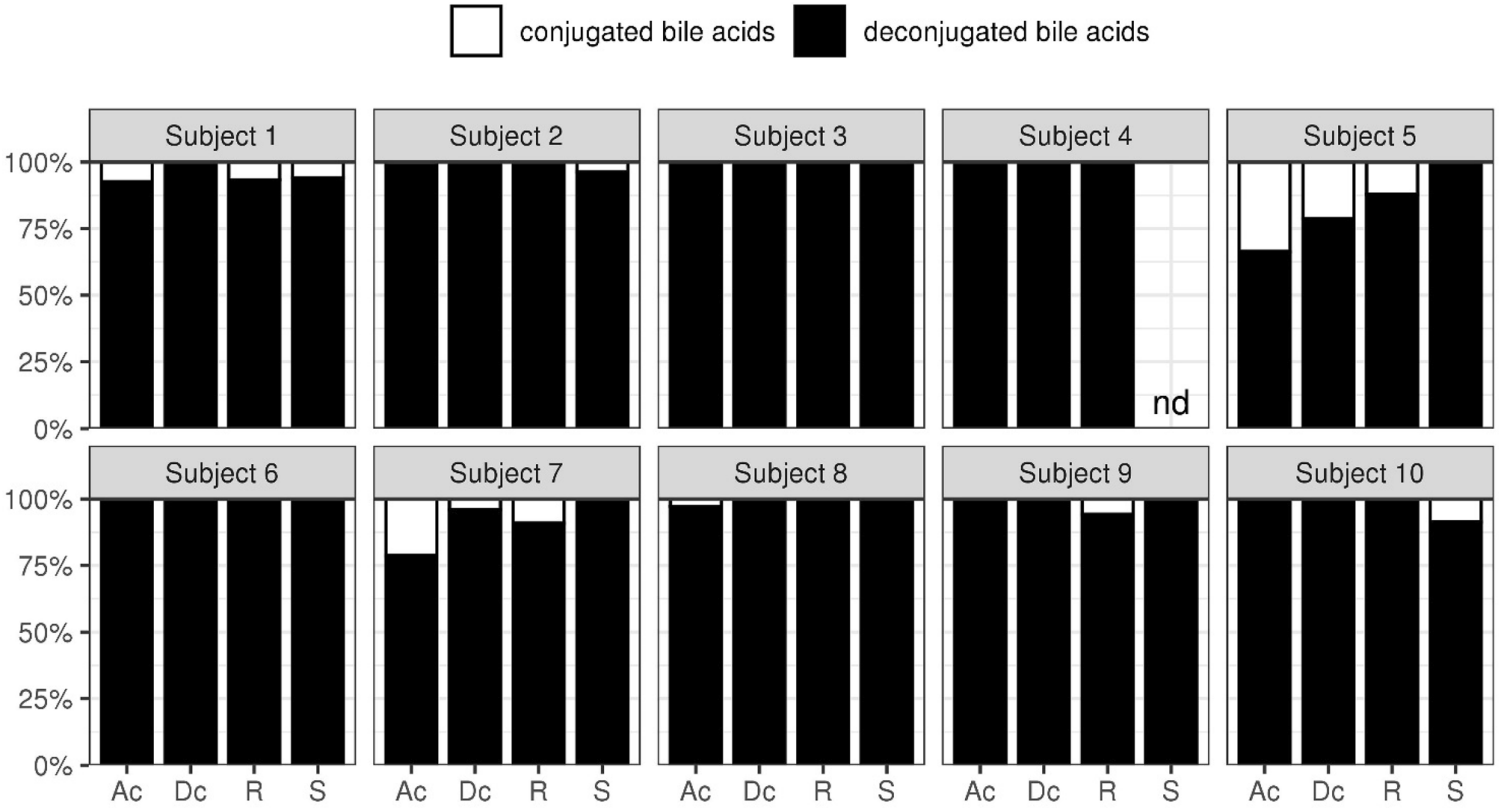
The deconjugation rate of bile acids in stool and contents from each part of the gut. The ratio (%) of deconjugated bile acids to the total bile acids’ concentration is represented. Ac: ascending colon; Dc: descending colon; R: rectum; S: stool.

**Figure 7:**
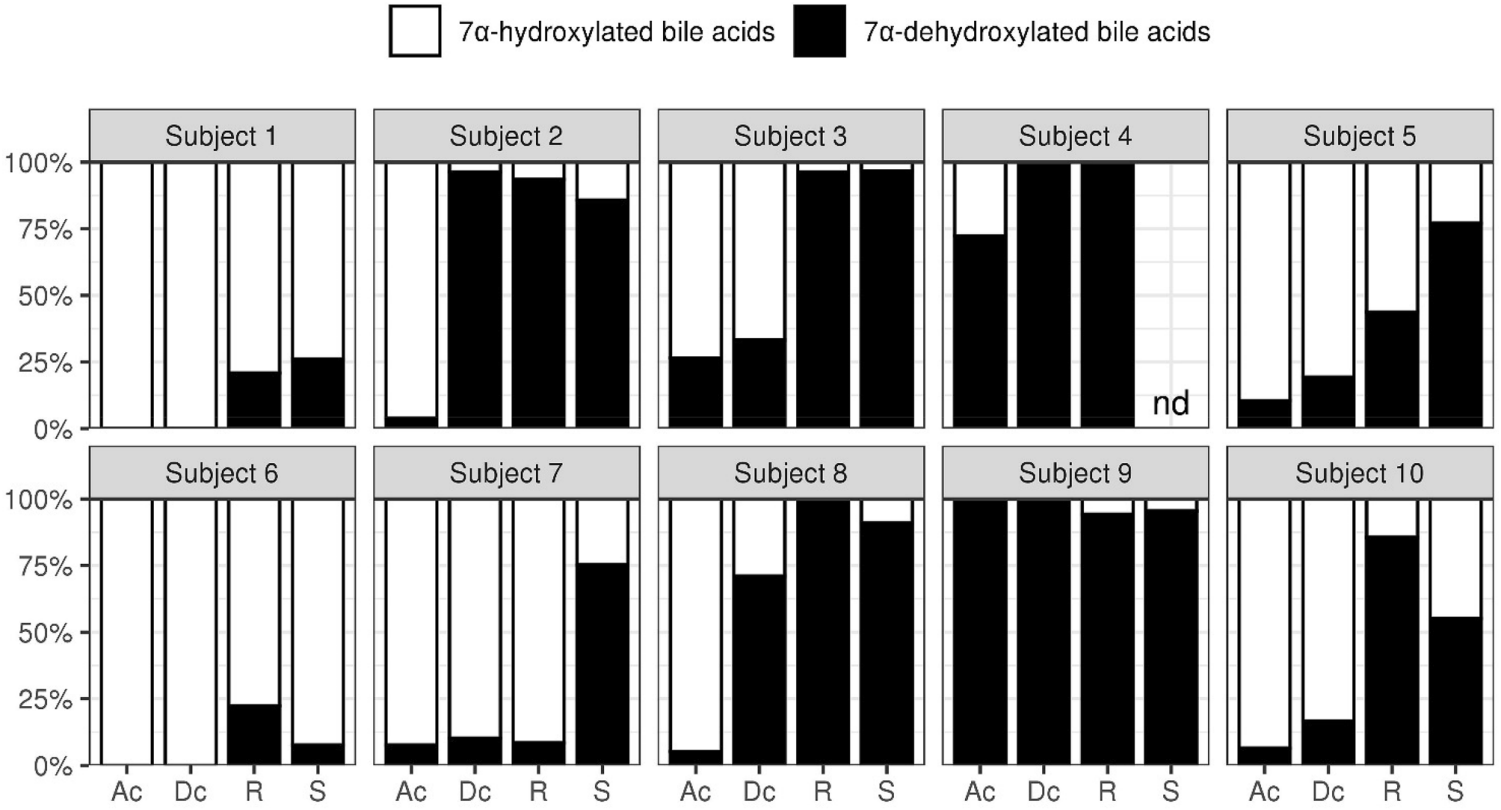
The 7α-dehydroxylation rate of bile acids in stool and contents from each part of the gut. The ratio (%) of 7α-dehydroxylated bile acids to the total bile acid concentration is represented. Ac: ascending colon; Dc: descending colon; R: rectum; S: stool.

The proportion of deconjugated and 7α-dehydroxylated bile acids were evaluated in gut contents and stool (Figs. 6 and 7). The proportion of deconjugated bile acids exceeded 90% in the ascending colon for 8 subjects. At these proportions, the reaction would have already completed in the upper colon. Only subjects 5 and 7 had the proportion of deconjugated bile acids of 80% in the descending colon. The maximum proportion of 7α-dehydroxylated bile acids in subjects 1 and 6 was around 20%, which was lower than the maximum proportion of 7α-dehydroxylated bile acids seen in other subjects (70% or more). Furthermore, while the reaction had been completed in the ascending colon for subjects 4 and 9, in other subjects the reaction tended to proceed, moving from the ascending colon to the rectum.

#### Inference of presumed bai-exhibiting bacteria from the feature sequences

**Figure 8:**
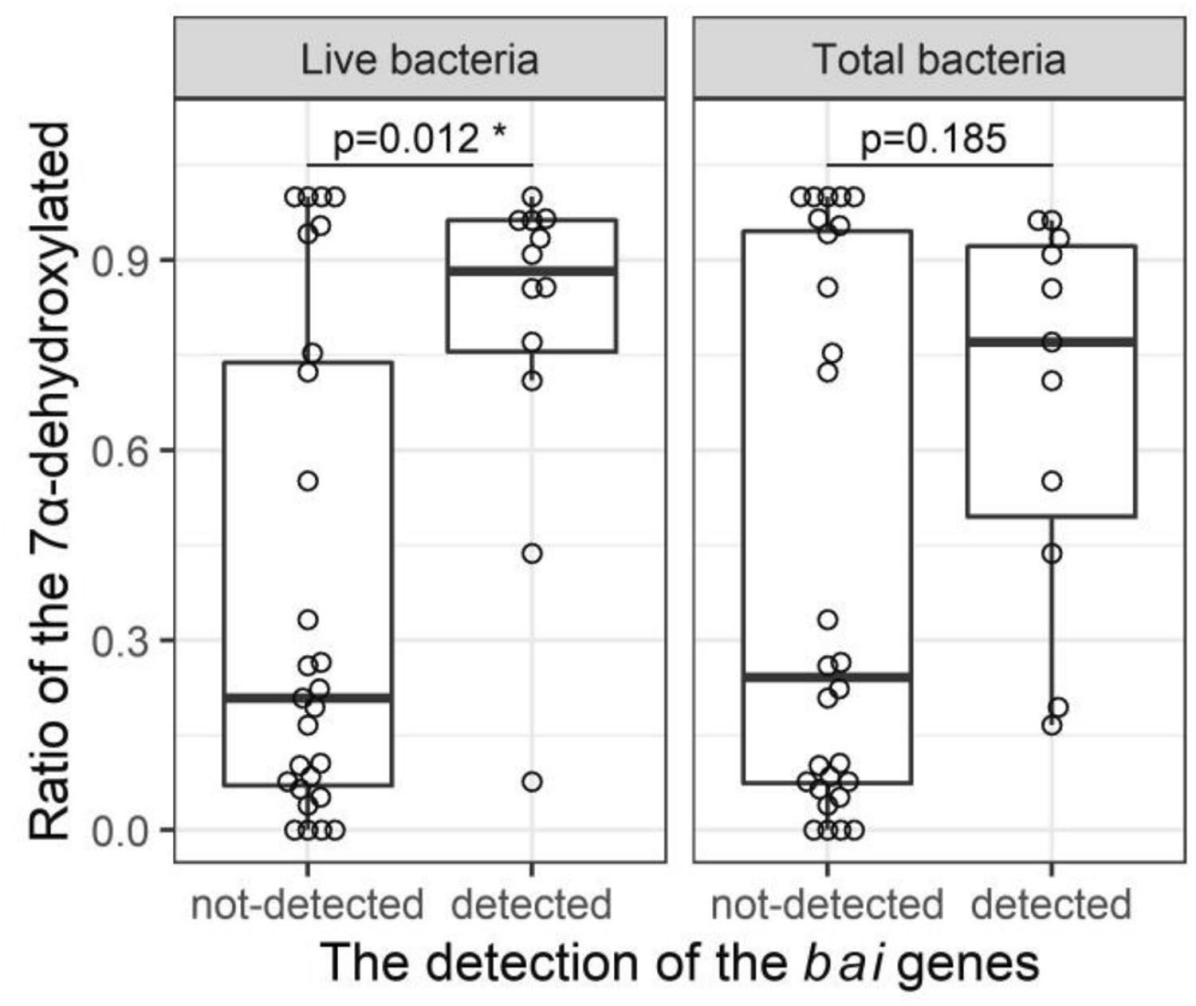
Relationship between 7α-dehydroxylation rate and the abundance of the presumed *bai*-exhibiting features in total and live bacteria. The box plots of 7α-dehydroxylation rates were used to investigate the relationship between 7α-dehydroxylation rate and the presence or absence of presumed *bai*-exhibiting features in total and live bacteria. Statistical analysis was performed using the Wilcoxon's rank-sum test: *P < 0.05.

We investigated the relationship between the rates of 7α-dehydroxylation and the presence or absence of the presumed *bai*-exhibiting features in total and live bacteria. Out of the 35 samples (Table 9) showing 7α-dehydroxylation, the presumed *bai-*exhibiting features were detected in 15 samples in both total and live bacteria (N = 8) and in total (N = 3) or live (N = 4) bacteria alone. All the presumed *bai-*exhibiting features were identified as *Clostridium scindens* (Supplementary Table 1); the mean read count of 16S rRNA detected was 3.0 (data not shown). For the remaining samples showing 7α-dehydroxylation (N = 20), no presumed *bai*-exhibiting features were detected (Table 9). Samples in which the presumed *bai-*exhibiting features were detected tended to show higher 7α-dehydroxylation rates and overall, the 7α-dehydroxylation rate was significantly higher in the presumed *bai-*exhibiting features detected group than that in the presumed *bai-*exhibiting features undetected group, especially in live bacteria (Fig. 8, *P* = 0.012). However, we could not detect the presumed *bai*-exhibiting features in eight samples: ascending colon, descending colon, and rectum of subject 4; stool of subject 7; and ascending colon, descending colon, rectum, and stool of subject 9, even though the 7α-dehydroxylation rates were high (70%–100%) in these samples.

**Table 9:** 7α-Dehydroxylation of bile acids and presumed *bai*-exhibiting features in stool and gut contents.

Subjects	Region	Totalbacteria	Livebacteria	Ratio of the 7α-dehydroxylated (%)
1	AC	−	−	0.0
1	DC	−	−	0.0
1	Rectum	−	−	20.9
1	Stool	−	−	26.0
2	AC	−	−	3.9
2	DC	+	+	96.3
2	Rectum	+	+	93.5
2	Stool	−	+	85.8
3	AC	−	−	26.5
3	DC	−	−	33.3
3	Rectum	+	+	96.3
3	Stool	−	+	96.6
4	AC	−	−	72.4
4	DC	−	−	100.0
4	Rectum	−	−	100.0
4	Stool	−	−	NT
5	AC	−	−	10.6
5	DC	+	−	19.4
5	Rectum	+	+	43.8
5	Stool	+	+	77.1
6	AC	−	−	0.0
6	DC	−	−	0.0
6	Rectum	−	−	22.3
6	Stool	−	+	7.7
7	AC	−	−	7.7
7	DC	−	−	10.2
7	Rectum	−	−	8.5
7	Stool	−	−	75.3
8	AC	−	−	5.2
8	DC	+	+	71.0
8	Rectum	−	+	100.0
8	Stool	+	+	90.9
9	AC	−	−	100.0
9	DC	−	−	100.0
9	Rectum	−	−	94.2
9	Stool	−	−	95.5
10	AC	−	−	6.5
10	DC	+	−	16.6
10	Rectum	+	+	85.6
10	Stool	+	−	55.1

*NT* not tested.

## Discussion

The gut microbiota consists of two populations within two different environments: LM and MAM^20,21^. Previous reports on LM and MAM from various gut sections focused on both live and dead bacteria together (total bacteria). Though considerable differences were detected between individual subjects, no clear differences in microbiota composition were identified in the context of different parts of the same individual’s gut^11,13,14,22,23^. In addition to conventional total bacteria microbiota analysis, the present study incorporated a PMA-MiSeq approach to elucidate the composition of only live bacteria.

Firstly, for LM, α-diversity analysis demonstrated that live bacteria tended to show lower diversity than total bacteria in all areas of the gut. If each OTU died at a similar rate, some rare strains would be non-detectable, resulting in a decrease in observed OTUs and PD, while the Shannon index, an index that considers both the abundance and evenness of OTUs, would be expected to remain almost unchanged. However, although the observed OTUs and PD in live LM did not significantly decrease, the Shannon index did decrease significantly. This suggests that the significant decrease in α-diversity observed in live LM was due to the dominance of specific species, such as *Bacteroidaceae and Lachnospiracea*, leading to a decrease in evenness. In fact, our results revealed that the relative abundance of live bacteria in LM from the same part of the gut showed high abundance of *Bacteroidaceae* and *Bifidobacteriaceae* and a low abundance of *Lachnospiraceae* compared to that of total bacteria composition for all subjects. Furthermore, the comparison of total bacteria and live bacteria abundance using DESeq2 analysis also showed that the abundance of OTUs in these three bacterial groups differed significantly. Considering these results, and the fact that there was no difference in the total LM abundance of the three bacterial groups among the three gut sites, we speculate that the growth of *Bacteroidaceae*, *Bifidobacteriaceae*, and *Lachnospiraceae* reaches the stationary phase in the ascending colon, and then *Lachnospiraceae* die off more rapidly than *Bacteroidaceae* and *Bifidobacteriaceae*. In other words, the difference in the abundance of each bacterial group between live and total LM is likely due to differences in the death rate before reaching the rectum. This is supported by our results, which demonstrate that the abundance of *Lachnospiraceae* in live LM gradually decreased in the order–ascending colon, descending colon, rectum, and stool– a fact that is reported for the first time by this study. Since many butyrate-producing bacteria belong to *Lachnospiraceae*, this result indicates that the ascending colon might be the primary site for the production of this short-chain fatty acid. Butyrate is an energy source for colonic mucosal epithelial cells, and its production by gut bacteria is crucial for the maintenance of host health. Although not achieved in our present study, this hypothesis could be further supported by comparing the composition of short-chain fatty acids among the three gut sites and stool and by analyzing the characteristics of isolates from each site. Of note, *Bacteroidetes* (including *Bacteroidaceae*) and *Firmicutes* (including *Lachnospiraceae*) are the most dominant bacterial groups accounting for more than 80% of the human gut microbiota^24^. The difference in composition between total bacteria (live and dead bacteria) and live bacteria is a very important finding in considering the interaction of the gut microbiota and physiological effects on the host. Since these two bacterial groups have a great influence on host health, this study, showing that their abundance within live bacteria assemblages differs depending on the part of the gut, is of great significance.

Secondly, for MAM, α-diversity analysis showed that the diversity of live bacteria was significantly lower than that of total bacteria in most parts of the gut in terms of the observed OTUs, PD, and Shannon index. The significant decrease in the three α-diversity metrics suggests that both the number of detectable strains and the evenness in live MAM were lower than those in the total. Therefore, we speculate that some specific species become dominant, while some other species become undetectable. Our results revealed that the abundance of *Enterobacteriaceae* in live MAM was eight times higher than its abundance in total composition from the same part of the gut. MAM is thought to impact epithelial and mucosal functions to a greater extent than LM. In the present study, the total MAM composition in the rectum differed from the composition in both the ascending and descending colons. Additionally, the total MAM composition did not differ significantly from those of the LM in the ascending and descending colons; differences were only seen in the rectum. These are likely explained by the difference in the thickness of the gut mucus layer between the colon sections and the rectum^25^. In the rectum, a thick mucus layer consisting of a dense inner layer and a low-density outer mucus layer covers the luminal surface of the epithelium; the majority of the gut microbiota is thought to be present in the outer mucus layer^26,27^. The mucus layers of the ascending colon and the descending colon are thin and fragile; therefore, suggesting that their MAM is strongly affected by LM. On the other hand, the live MAM composition differed significantly from the live LM at all three gut sites, with particularly higher abundance of facultative anaerobe *Enterobacteriaceae*. We suggest that this can be explained by the presence of a spatial oxygen gradient^28^. Thus, our present study reveals that the rectum MAM has a unique microbiota.

Bile acids play an important role in the digestion and absorption of lipids; particularly, they suppress the overgrowth of intestinal bacteria in the upper digestive tract^29^. Here, we investigated the bile acid composition in each part of the colon, focusing on the capacity of intestinal bacteria to drive deconjugation and 7α-dehydroxylation reactions. We found that deconjugation is completed in the ascending colon, while 7α-dehydroxylation proceeds as bile acids move from the ascending colon to the rectum. Based on 3 forensic autopsy cases, Ishimoto reported that the proportion of deconjugated bile acids in the terminal ileum was approximately 80%; moreover, deconjugation was highest in samples cultured anaerobically, suggesting that anaerobic bacteria are the primary drivers^30^. Our results are consistent with this report; therefore, we envision that deconjugation of bile acids progresses rapidly from the distal ileum to the ascending colon with the help of anaerobic bacteria. We have previously investigated the proportion of deconjugated bile acids in ileal fluid from healthy adults and showed that the mean proportion of deconjugated bile acids was approximately 20%^31^. The resident bacterial count in the proximal ileum, where aerobic/facultative anaerobic bacteria predominate (10^7^ cells/g), is much lower than that in the colon, where anaerobic bacteria are predominant (10^12^ cells/g)^32^. In other words, when bile acids are secreted, the low bacterial counts do not support their deconjugation that proceeds only in the terminal ileum/colon, where bacterial counts increase.

Deoxycholic acid and lithocholic acid, which are 7α-dehydroxylated bile acids, have potent cytotoxic activity and are involved in carcinogenesis^17^, and in the inhibition of *Clostridium difficile*^33^*. Clostridium scindens* is known for their strong 7α-dehydroxylation activity^34^; *bai* genes within one operon (8 gene clusters) have been associated with this activity^35^, and attempts have been made to elucidate the metabolic pathway^36^. We investigate the relationship between the rates of 7α-dehydroxylation and the abundance of the presumed *bai*-exhibiting features in total and live bacteria. Importantly, all of the presumed *bai*-exhibiting features detected in this study were related to *C. scindens*. However, the mean abundance of this bacterial group was 0.02% of total bacteria, similar to that in previous reports^37^. In live bacteria, the 7α-dehydroxylation rate was higher in the presumed *bai*-exhibiting features detected group than in the group where the presumed *bai*-exhibiting features were not detected. This suggests that 7α-dehydroxylation is directly linked to viability of *C. scindens*. Surprisingly, subjects 4 and 9 showed high 7α-dehydroxylation rates in all samples, but no presumed *bai*-exhibiting features were detected, suggesting that the bacterial species responsible for 7α-dehydroxylation was not very abundant and, therefore, below the detection limit of our technique. However, this could also indicate the presence of an unknown bacterial species are involved in 7α-dehydroxylation. Vital et al. (2019) used genomes of isolates and metagenome-assembled genomes to investigate human gut bacterial species involved in 7α-dehydroxylation and reported that many bacterial species yet to be isolated are involved in 7α-dehydroxylation^38^. In this study, we could not obtain the V1-V2 regions of 16S rRNA from the group closely related *Firmicutes bacterium* CAG:103^38^, which is the majority of a presumed *bai*-exhibiting bacteria. Going forward, it would be necessary to isolate the unidentified bacteria involved in 7α-dehydroxylation and test 7α-dehydroxylation capacity in vitro.

One limitation of this study is the low sample size. Nishijima et al. (2016) have pointed out that the gut microbiota of Japanese populations are vastly different from gut microbiota of populations from other regions and countries^39^. In the present study, all subjects were adult males in their 20 s, meaning that age and sex differences were not investigated. Takagi et al. (2019) have reported that while there is no difference in the α-diversity of microbiota structure between Japanese men and women, there is a significant difference in terms of α-diversity^40^. Moreover, differences in the microbiota between infants, adults and elderly people were also reported^41,42^. In the future, further analyses using larger sample sizes and different regions, genders, and ages will be necessary.

To date, analysis of gut microbiota has predominantly used stool samples, and discussions related to host health have been based on results of total bacteria in the stool, including dead ones. However, information obtained from stool alone is not enough to understand the metabolic activity of intestinal bacteria essential for the promotion of human health; it is essential to analyze both live bacteria and subsequent metabolites in various parts of the intestinal tract to understand fully the impact of the gut microbiota.

## Conclusion

The present study demonstrated that there are differences in the composition of total bacteria and live bacteria in different parts of the human gut and that PMA-MiSeq is useful for the analysis of live bacteria. In addition, it also showed that differences in composition of live bacteria assemblages in each part of the gut may be one of the factors affecting the metabolism of bile acids. The presence of an unknown bacterial species involved in 7α-dehydroxylation was also indicated. Many things remain unclear about how dead and live bacteria affect the human body. However, in order to elucidate metabolomics fully, it is necessary to analyze live gut microbiota.

## Methods

### Subjects and ethics

Ten healthy male volunteers (21.2 ± 0.9 years old) participated in this study. They were asked to restrict intake of fermented milk, fermented foods, and probiotic-containing foods for one week prior to the study date. They were also instructed to eat the colonoscopy/CT test food “FG-two☆” (Fushimi Pharmaceutical Co., Ltd.) instead of normal food for two days before the study. On the day before or on the day of the study, they were asked to sample part of their stool. To make sure that subjects had residual gut contents for the study, they were not subjected to common colonoscopy interventions such as use of laxatives. When the residual stools were evaluated on the 5-point (Excellent, Good, Fair, Poor, Inadequate) Aronchick Bowel Preparation Scale^43^ during colonoscopy, the “inadequate” condition was not seen in any subjects. Therefore, the colonoscope could be inserted without any problem. This study was conducted based on the ethical principles of the Declaration of Helsinki and was approved by the Hirosaki University Graduate School of Medicine Ethics Committee (ID: 2016–006). The subjects’ personal information was anonymized; care was taken not to disclose such information. The subjects were informed about the purpose and risks of the study before they were asked to provide written consent.

### Sampling methods

On the day before the study, all subjects collected 0.5 g of stool sample into a stool specimen container (Sarstedt AG & Co. KG [cat. no. 80.734.001]). All samples were immediately stored at 4 °C in aerobic condition after collection. Each stool sample was dispersed directly into 1 mL PBS solution in an Eppendorf tube via immersion and shaking, and appropriate amount of PBS was then added to obtain a total volume of 1.2 mL or more. Then, 2 µL of 5 mM PMAxx™ (Biotium, Inc. [cat. no. #40019]) was added and mixed with 200 µL of the stool sample suspension; the mixture was allowed to stand for 5 min on ice away from light and then irradiated for 10 min using LED Crosslinker 12 (Takara Bio, Inc. [cat. no. EM200]). Subsequently, the mixture was centrifuged, the supernatant was removed, and the pellets were frozen in dry ice. The remaining sample suspension was similarly frozen and stored at − 80 °C until further use. The different parts of the gut were sampled, from the rectum to the descending and then the ascending colons; mucus and gut contents were collected. To sample the gut contents, we used a self-made instrument (Supplementary Fig. 1). Briefly, the tip of a fluoresin dye-dispersion tube (Endoshower; Yasec, Shiga, Japan [25B2X10005000001]), slightly thinner than the size of the forceps hole (2.8 mm), was plugged with beeswax (Alfresa Pharma, Osaka, Japan [274889172]) to prevent contamination and passed through the biopsy forceps Radial Jaw 4P (Boston Scientific, Tokyo, Japan [13B1X00043000043]). We also used a self-made instrument to sample the mucus, wherein the tip of an RX Cytology Brush (Boston Scientific, Tokyo, Japan [13B1X00043000027]) was plugged with beeswax. A new sampling instrument was used for each part of the gut sampled. The arrival of the instrument to the sampling site was confirmed using X-ray fluoroscopy. The sampling instruments were inserted through the colonoscope forceps hole as follows. We first inserted the scrape cytology brush and pressed it strongly against the mucosal tissue, which did not appear to have any gut contents adhered to it, before scraping the area 10 times to obtain a mucosal tissue sample. The sample was deposited directly into 1 mL PBS solution in an Eppendorf tube via immersion and shaking. The gut contents were then sampled using the biopsy forceps. A sterilized toothpick was used to transfer the sampled contents to a pre-tared Eppendorf tube. This step was repeated thrice before the weight of gut contents was measured and appropriate amount of PBS was added to obtain a total volume of 1.2 mL or more. PMA treatment, photoactivation, and preservation of the gut content and the mucus sample suspension were performed in a manner similar to that regarding stool samples described above.

### α-Diversity analysis

α-Diversity analysis was performed using QIIME2 ver. 2019.7^44^. The α-diversity indicators were calculated using the diversity core-metrics-phylogenetic plugin (sampling depth 5000).

### Microbiota analysis

All samples were stabilized using RNA*later*®. PMA-treated and untreated stool, gut content, and mucus samples were each washed twice with PBS prior to DNA extraction. DNA was extracted using cell disruption glass beads and phenol, as previously reported^45^. As per a previous report^46^, the V1-V2 regions of the 16S rRNA gene were amplified using 27Fmod2-MiSeq and 338R-MiSeq as primers. Amplification was performed as follows: 2 min at 50 °C, 10 min at 95 °C, 30 s at 95 °C, 30 s at 55 °C and finally, 90 s at 72 °C; the cycle was repeated until the reaction plateaued. The resulting MiSeq library was analyzed using the MiSeq Reagent Kit v2 (Illumina [cat. no. MS-102–2001]). The sequence data was then analyzed using QIIME2^44^; the DADA2^47^ plugin was used to remove noise and trim the sequences. Processing via DADA2 was performed separately each time MiSeq was run with the same parameters; the features table and sequence were merged. The feature sequence was then classified using classify-sklearn. The classifier was trained on the Greengenes database (13_8 release; 99% identity clusters) using the fit-classifier-native-bayes function. The features were annotated using vsearch^48^ and the NCBI database 16S RefSeq (BioProject IDs: 33,175 and 33,317, downloaded on 11 January 2019). We performed OTU clustering by using the R package DECIPHER^49^ functions. The features produced by DADA2 in QIIME2 were aligned by using AlignSeqs. A distance matrix was built from the aligned data by using DistanceMatrix; this matrix was used to identify clusters by using IdClusters with the UPGMA method and a cutoff value of 0.03 to make 97% OTUs. OTU representative sequences were selected by the largest count feature in the OTU and were annotated by using vsearch as described above.

### Bile acid quantification

Diluted gut content and stool solutions were lyophilized before adding 1 mM 5β-Pregnan-3α, 17α, 20α-triol (pregnanetriol: M.W. 336.5, Sigma [cat. no. P8629])-methanol solution as an internal standard. After the addition of 99.5% ethanol, the mixture was subjected to 2 h of thermal extraction at 70 °C and then centrifuged (304 × *g*, 15 min). After extraction, the supernatant was evaporated to dryness, and the residual was dissolved in an appropriate amount of methanol. This solution was then filtered and used as a sample for the analysis of bile acids. The bile acid composition was analyzed via high performance liquid chromatography (HPLC)^50^, passing a 0.1 mM standard substance (Glycoursodeoxycholic acid [GUDCA], Tauroursodeoxycholic acid [TUDCA], Ursodeoxycholic acid [UDCA], Glycocholic acid [GCA], Taurocholic acid [TCA], Cholic acid [CA], Glycochenodeoxycholic acid [GCDCA], Taurochenodeoxycholic acid [TCDCA], Glycodeoxycholic acid [GDCA], Taurodeoxycholic acid [TDCA], Chenodeoxycholic acid [CDCA], Deoxycholic acid [DCA], Glycolithocholic acid [GLCA], Taurolithocholic acid [TLCA], Lithocholic acid [LCA])-mixed solution (Supplementary Fig. 2) and each sample through a reversed-phase C18 column (Bile PakII, JASCO [code no. D511]) and a 3α-HSD (3 α-hydroxysteroid dehydrogenase)-immobilized enzyme column (JASCO [code no. D512]).

### Inference of presumed bai-exhibiting bacteria from the feature sequences

Vital et al. (2019) reported a comprehensive overview of the diversity of *bai*-exhibiting bacteria in the human gut by screening genomes of isolates and metagenome-assembled genomes (MAGs)^38^. Referencing this study, we downloaded *bai*-gene-containing/non-*bai*-gene-containing genomes. Then, we extracted the V1-V2 regions of the 16S rRNA gene (27Fmod2-MiSeq and 338R-MiSeq as primers) as reference sequences of *bai*-exhibiting/non-*bai*-exhibiting bacteria (Supplementary table 2, supplementary fasta file). Notably, we excluded the V1-V2 regions from an assembly (accession: GCF_900074625.1) since it contained too many sequence variants (10 variants), suggesting sequencing error. The features produced by DADA2 in QIIME2 were annotated using vsearch^48^ and the reference sequences as database. Based on the annotation results, we presumed the features which showed > 95% similarity to reference sequences were from *bai*-exhibiting bacteria and those showing < 90% similarity were from non-*bai*-exhibiting bacteria (Supplementary Table 1). Additionally, we produced a box-plot showing the rates of 7α-dehydroxylation to investigate the relationship between 7α-dehydroxylation and the presence/absence of the presumed *bai*-exhibiting features in total and live bacteria (Fig. 8).

### Statistical analysis

All statistical analyses were conducted in R (version 4.0.5; http://cran.r-project.org/). Bacterial viability rates among the three gut sites (ascending colon, descending colon, and rectum) and stool were compared using Friedman test. We performed a permutational multivariate analysis of variance (PERMANOVA, adonis function in the R package vegan)^51^ based on the Bray–Curtis distance to test whether the bacterial community composition differed significantly between categorical groups. The test was run with 9,999 permutations. The Bray–Curtis distance matrix was based on relative abundance of taxa at the family level and was calculated by the vegdist function in the R package vegan. We performed NMDS ordination with the Bray–Curtis distance described above by using vegan function metaMDS. Pairwise comparisons using PERMANOVA were conducted and corrected for multiple testing by using the Benjamini–Hochberg correction as implemented in the pairwise.perm.manova function in the R package RVAideMemoire^52^.

We used DESeq2’s^53^ likelihood ratio test (LRT) to identify taxa that differed significantly in abundance across categorical variables. In the LRT, we compared two models with and without the terms of interest, and the models were specified in each analysis. Raw read counts were normalized by geometric mean of pairwise ratios (GMPR)^54^. For normalization, we estimated the size factors on the basis of the OTU-level data. The log2 fold changes were shrunk by using the DESeq2 function lfcShrink with the apeglm^55^ shrinkage estimator type. Taxa for which the Benjamini–Hochberg adjusted *p*-value was < 0.05 were considered to be statistically significant. Wilcoxon's rank-sum test was used to compare the results of α-diversity between total and live bacteria in each part of the gut, as well as between LM and MAM. The 7α-dehydroxylation rates were compared between the detected and undetected groups of *bai* genes using Wilcoxon's rank-sum test within each group of total and live bacteria.

### Patient and public involvement

Patients and/or the public were not involved in the design, or conduct, or reporting or dissemination plans of this research**.**

### Patient consent for publication

Not required.

### Ethics approval

This study was conducted based on the ethical principles of the Declaration of Helsinki and was approved by the Hirosaki University Graduate School of Medicine Ethics Committee (ID: 2016-006).

## Supplementary Information

Supplementary Information 1.

Supplementary Information 2.

## Data Availability

The dataset used in this study is publicly available through the DNA Data Bank of Japan (DDBJ) DDBJ Sequence Read Archive (DRA) under accession number PRJDB10649.
