# Supplementary material for: Spatial distribution of live gut microbiota and bile acid metabolism in various parts of human large intestine
Source: Sci Rep. 2022 Mar 4;12:3593. doi: 10.1038/s41598-022-07594-6 (PMC8897406; doi:10.1038/s41598-022-07594-6)
Supplement: Supplementary file 1 — Supplementary Information 1. [file 41598_2022_7594_MOESM1_ESM.docx]

>ref01

GACGAACGCTGGCGGCGTGCCTAACACATGCAAGTCGAACGAAGCTCGATGCTTTGAGGTTTCGGCCAAGAAGCAAAGATGACTTAGTGGCGAACGGGTGAGTAATGCGTGAGCAACCTGCCTTTCAGAGGGGGACAACAGCTGGAAACGGCTGCTAATACCGCATAATACATTAGGGTGGCATCACCTTGATGTCAAAGGAGCAATCCGCTGAGAGATGGGCTCACGTCTGATTAGCTAGTTGGTGAGGTAACGGCTCACCAAGGCGACGATCAGTAGCCGGTCTGAGAGGATGAACGGCCACATTGGGACTGAGATACGGCCCAGACTCCTACGGGAGGCAG

>ref02

GAGTTTGATCCTGGCTCAGGACGAACGCTGGCGGCGTGCCTAACACATGCAAGTCGAACGAAGCTCGATGCTTTGAGGTTTCGGCCAAGAAGCAAAGATGACTTAGTGGCGAACGGGTGAGTAATGCGTGAGCAACCTGCCTTTCAGAGGGGGACAACAGCTGGAAACGGCTGCTAATACCGCATAACACATTGGGGTGGCATCACCTTGATGTCAAAGGAGCAATCCGCTGAGAGATGGGCTCACGTCTGATTAGCTAGTTGGTGAGGTAACGGCTCACCAAGGCGACGATCAGTAGCCGGTCTGAGAGGATGAACGGCCACATTGGGACTGAGATACGGCCCAG

>ref03

GAGTTTGATCCTGGCTCAGGACGAACGCTGGCGGCGTGCCTAACACATGCAAGTCGAACGAAGCTTGAAGCTTTGAGATTTCGGTCAAGAAGCGGATATGACTTAGTGGCGAACGGGTGAGTAATGCGTGAGCAACCTGCCTTTCAGAGGGGGACAACAGCTGGAAACGGCTGCTAATACCGCATAACACATTTGGGTGGCATCACCTGGATGTCAAAGGAGCAATCCGCTGAGAGATGGGCTCACGTCTGATTAGCTAGTTGGTGAGGTAACGGCTCACCAAGGCGACGATCAGTAGCCGGTCTGAGAGGATGAACGGCCACATTGGGACTGAGATACGGCCCAG

>ref04

GAGTTTGATCCTGGCTCAGGACGAACGCTGGCGGCGTGCTTAACACATGCAAGTCGAACGGAGCACCCTTGACTGAGGTTTCGGCCAAATGATAGGAATGCTTAGTGGCGGACTGGTGAGTAACGCGTGAGGAACCTGCCTTCCAGAGGGGGACAACAGTTGGAAACGACTGCTAATACCGCATGACGCATGACCGGGGCATCCCGGGCATGTCAAAGATTTTATCGCTGGAAGATGGCCTCGCGTCTGATTAGCTAGATGGTGGGGTAACGGCCCACCATGGCGACGATCAGTAGCCGGACTGAGAGGTTGACCGGCCACATTGGGACTGAGATACGGCCCAG

>ref05

GAGTTTGATCCTGGCTCAGGATGAACGCTGGCGGCGTGCCTAACACATGCAAGTCGAACGAAGCGCCTGGCCCCGACTTCTTCGGAACGAGGAGCCTTGCGACTGAGTGGCGGACGGGTGAGTAACGCGTGGGCAACCTGCCTTGCACTGGGGGATAACAGCCAGAAATGGCTGCTAATACCGCATAAGACCGAAGCGCCGCATGGCGCGGCGGCCAAAGCCCCGGCGGTGCAAGATGGGCCCGCGTCTGATTAGGTAGTTGGCGGGGTAACGGCCCACCAAGCCGACGATCAGTAGCCGACCTGAGAGGGTGACCGGCCACATTGGGACTGAGACACGGCCCAG

>ref06

GAGTTTGATCCTGGCTCAGGATGAACGCTGGCGGCGTGCCTAACACATGCAAGTCGAGCGAACCCTTCGGGGTGAGCGGCGGACGGGTGAGTAACGCGTGGGTAACCTGCCCTGTACACACGGATAACATACCGAAAGGTATGCTAATACGGGATAACATATGAGAGTCGCATGGCTTTTGTATCAAAGCTCCGGCGGTACAGGATGGACCCGCGTCTGATTAGCTAGTTGGTAAGGTAACGGCTTACCAAGGCAACGATCAGTAGCCGACCTGAGAGGGTGATCGGCCACATTGGAACTGAGACACGGTCCAA

>ref07

GAGTTTGATCCTGGCTCAGGATGAACGCTGGCGGCGTGCCTAACACATGCAAGTCGAGCGATCTCTTCGGAGAGAGCGGCGGACGGGTGAGTAACGCGTGGGTAACCTGCCCTGTACACACGGATAACATACCGAAAGGTATACTAATACGGGATAACATACGAAAGTCGCATGGCTTTTGTATCAAAGCTCCGGCGGTACAGGATGGACCCGCGTCTGATTAGCTAGTTGGTAAGGTAATGGCTTACCAAGGCAACGATCAGTAGCCGACCTGAGAGGGTGATCGGCCACACTGGAACTGAGACACGGTCCAG

>ref08

GAGTTTGATCCTGGCTCAGGATGAACGCTGGCGGCGTGCCTAACACATGCAAGTCGAGCGATCTCTTCGGAGAGAGCGGCGGACGGGTGAGTAACGCGTGGGTAACCTGCCCTGTACACACGGATAACATACCGAAAGGTATGCTAATACGGGATAATATATAAGAGTCGCATGACTTTTATATCAAAGATTTTTCGGTACAGGATGGACCCGCGTCTGATTAGCTTGTTGGCGGGGTAACGGCCCACCAAGGCGACGATCAGTAGCCGACCTGAGAGGGTGATCGGCCACATTGGAACTGAGACACGGTCCAA

>ref09

GAGTTTGATCCTGGCTCAGGATGAACGCTGGCGGCGTGCCTAACACATGCAAGTCGAGCGATTCTCTTCGGAGAAGAGCGGCGGACGGGTGAGTAACGCGTGGGTAACCTGCCCTGTACACACGGATAACATACCGAAAGGTATGCTAATACGAGATGATATATTTTTATTGCATGATAAAAATATCAAAGCTTTTGCGGTACAGGATGGACCCGCGTCTGATTAGCTAGTTGGTAAGGTAACGGCTTACCAAGGCGACGATCAGTAGCCGACCTGAGAGGGTGATCGGCCACACTGGAACTGAGACACGGTCCAG

>ref10

GAGTTTGATCCTGGCTCAGGATGAACGCTGGCGGCGTGCCTAACACATGCAAGTCGAGCGATTCTCTTCGGAGAAGAGCGGCGGACGGGTGAGTAACGCGTGGGTAACCTGCCCTGTACACACGGATAACATACCGAAAGGTATGCTAATACGGGATGATATATAAGAGTCGCATGACTTTTATATCAAAGATTTTTCGGTACAGGATGGACCCGCGTCTGATTAGCTTGTTGGCGGGGTAACGGCCCACCAAGGCGACGATCAGTAGCCGACCTGAGAGGGTGATCGGCCACATTGGAACTGAGACACGGTCCAA

>ref11

GAGTTTGATCCTGGCTCAGGATGAACGCTGGCGGCGTGCCTAACACATGCAAGTTGAGCGATTTACTTCGGTAAAGAGCGGCGGACGGGTGAGTAACGCGTGGGTAACCTACCCTGTACACACGGATAACATACCGAAAGGTATGCTAATACGGGATAATATATTTGAGAGGCATCTCTTGAATATCAAAGGTGAGCCAGTACAGGATGGACCCGCGTCTGATTAGCTAGTTGGTAAGGTAACGGCTTACCAAGGCGACGATCAGTAGCCGACCTGAGAGGGTGATCGGCCACATTGGAACTGAGACACGGTCCAA

>ref12

GAGTTTGATCCTGGCTCAGGATGAACGCTGGCGGCGTGCTTAACACATGCAAGTCGAACGAAGCACTTAAGTTTGATTCTTCGGATGAAGACTTTTGTGACTGAGTGGCGGACGGGTGAGTAACGCGTGGGTAACCTGCCTCATACAGGGGGATAACAGTTAGAAATGACTGCTAATACCGCATAAGACCACAGCACCGCATGGTGCAGGGGTAAAAACTCCGGTGGTATGAGATGGACCCGCGTCTGATTAGCTAGTTGGTGGGGTAACGGCCTACCAAGGCGACGATCAGTAGCCGACCTGAGAGGGTGACCGGCCACATTGGGACTGAGACACGGCCCAA

>ref13

GAGTTTGATCCTGGCTCAGGATGAACGCTGGCGGCGTGCTTAACACATGCAAGTCGAACGAAGCACTTTTGATTGATTTCTTCGGAAAGAGAGAGACTGTGACTGAGTGGCGGACGGGTGAGTAACGCGTGGGTAACCTGCCTCATACAGGGGGATAACAGTTAGAAATGACTGCTAATACCGCATAAGCACACAGCTTCGCATGAAGCAGTGTGAAAAACTCCGGTGGTATGAGATGGACCCGCGTCTGATTAGGTAGTTGGTGGGGTAACGGCCCACCAAGCCGACGATCAGTAGCCGACCTGAGAGGGTGACCGGCCACATTGGGACTGAGACACGGCCCAA

>ref14

GAGTTTGATCCTGGCTCAGGATGAACGCTGGCGGCGTGCTTAACACATGCAAGTCGAACGGAGTGCCAATGACAGAGGATTCGTCCAATGGATTTGGTTACTTAGTGGCGGACGGGTGAGTAACGCGTGAGGAACCTGCCTTGGAGTGGGGAATAACACAGTGAAAATTGTGCTAATACCGCATAATGCAGTTGGGTCGCATGGCTCTGACTGCCAAAGATTTATCGCTCTGAGATGGCCTCGCGTCTGATTAGCTAGTTGGCGGGGTAACGGCCCACCAAGGCGACGATCAGTAGCCGGACTGAGAGGTTGGCCGGCCACATTGGGACTGAGACACGGCCCAG

>ref15

GAGTTTGATCCTGGCTCAGGATGAACGCTGGCGGCGTGCTTAACACATGCAAGTCGAGCGAAGCACTTAAGTTCGATTCTTCGGATGAAGACTTTTGTGACTGAGCGGCGGACGGGTGAGTAACGCGTGGGTAACCTGCCTCATACAGGGGGATAACAGTTAGAAATGGCTGCTAATACCGCATAAGACCACAGTACTGCATGGTACAGTGGTAAAAACTCCGGTGGTATGAGATGGACCCGCGTCTGATTAGGTAGTTGGTGAGGTAACGGCCCACCAAGCCGACGATCAGTAGCCGACCTGAGAGGGTGACCGGCCACATTGGGACTGAGACACGGCCCAG

>ref16

GAGTTTGATCCTGGCTCAGGATGAACGCTGGCGGCGTGCTTAACACATGCAAGTCGAGCGAAGCGCCTTATACAGATTCCTTCGGGATGAAGGATTTGGCGACTGAGCGGCGGACGGGTGAGTAACGCGTGGGCAACCTGCCTCACACAGGGGGATAACAGCTAGAAATGGCTGCTAATACCGCATAAGGCCGCGGCACCGCATGGTGCGGCGGCAGAAGCTCCGGCGGTGTGAGATGGGCCCGCGTCTGATTAGGTAGTTGGTGCGGTAACGGCGCACCAAGCCGACGATCAGTAGCCGACCTGAGAGGGTGACCGGCCACATTGGGACTGAGACACGGCCCAA

>ref17

GAGTTTGATCCTGGCTCAGGATGAACGCTGGCGGCGTGCTTAACACATGCAAGTCGAGCGAAGCGCTCTGTTCAGATTTCTTCGGAATGAAGAGCATTGCGACTGAGCGGCGGACGGGTGAGTAACGCGTGGGCAACCTGCCTCACACAGGGGGATAACAGCTAGAAATGGCTGCTAATACCGCATAAGGCTACAGAGCCGCATGGCTCGGTGGCAAAAGCTCCGGCGGTGTGAGATGGGCCCGCGTCTGATTAGGTAGTTGGTGGGGCAGAGGCCCACCAAGCCGACGATCAGTAGCCGACCTGAGAGGGTGACCGGCCACATTGGGACTGAGACACGGCCCAA

>ref18

GATGAACGCTGGCGGCATGCCTAACACATGCAAGTCGAACGGAGCACCCCTGACAGAGACTTCGGTCAATGGATGGGAATGCTTAGTGGCGGACGGGTGAGTAACGCGTGAGGAACCTGCCTTTCAGTGGGGGATAACACATCGAAAGATGTGCTAATACCGCATAACATAGCGATGGGGCATCCCATTGCTATCAAAGATTTATTGCTGAAAGATGGCCTCGCGTCCAATTAGCTAGTTGGTGAGGTAACGGCCCACCAAGGCGACGATTGGTAGCCGGACTGAGAGGTTGAACGGCCACATTGGGACTGAGACACGGCCCAGACTCCTACGGGAGGCAG

>ref19

GATGAACGCTGGCGGCATGCCTAACACATGCAAGTCGAACGGAGCACCCCTGACAGAGGCTTCGGCCAATGGAAGGGAATGCTTAGTGGCGGACGGGTGAGTAACGCGTGAGGAACCTGCCTTTCAGTGGGGGATAACACATCGAAAGATGTGCTAATACCGCATAACATAGCGATGGGGCATCCCATTGCTATCAAAGATTTATTGCTGAAAGATGGCCTCGCGTCCAATTAGCTAGTTGGTGAGGTAACGGCCCACCAAGGCGACGATTGGTAGCCGGACTGAGAGGTTGAACGGCCACATTGGGACTGAGACACGGCCCAGACTCCTACGGGAGGCAG

>ref20

GATGAACGCTGGCGGCGTGCCTAACACATGCAAGTCGAACGAAGCAATAAAGACGGAGATTCGTCAAAGCCTTTATTGACTTAGTGGCGGACGGGTGAGTAACGCGTGAGCAACCTGCCTTTCAGAGGGGGACAACAGTTGGAAACGACTGCTAATACCGCATAATATACAGAGGGGGCATCCCATTTGTATCAAAGGAGAAATCCGCTGAAAGATGGGCTCACGTCTGATTAGCTAGTTGGTGGGGTAAAGGCCTACCAAGGCTGCGATCAGTAGCCGGACTGAGAGGTTGAACGGCCACATTGGGACTGAGATACGGCCCAGACTCCTACGGGAGGCAG

>ref21

GATGAACGCTGGCGGCGTGCCTAACACATGCAAGTCGAACGAAGCGCCTGGCCCCGACTTCTTCGGAACGAGGAGCCTTGCGACTGAGTGGCGGACGGGTGAGTAACGCGTGGGCAACCTGCCTTGCACTGGGGGATAACAGCCAGAAATGGCTGCTAATACCGCATAAGACCGAAGCGCCGCATGGCGCGGCGGCCAAAGCCCCGGCGGTGCAAGATGGGCCCGCGTCTGATTAGGTAGTTGGCGGGGTAACGGCCCACCAAGCCGACGATCAGTAGCCGACCTGAGAGGGTGACCGGCCACATTGGGACTGAGACACGGCCCAGACTCCTACGGGAGGCAG

>ref22

GATGAACGCTGGCGGCGTGCTTAACACATGCAAGTCGAACGAAGCAATACTGTGTGAAGAGATTAGCTTGCTAAGATCAGAACTTTGTATTGACTGAGTGGCGGACGGGTGAGTAACGCGTGGGCAACCTGCCTTACACAGGGGGATAACAGCTAGAAATGGCTGCTAATACCGCATAAGACCTCAGTACCGCATGGTAGAGGGGTAAAAACTCCGGTGGTGTAAGATGGGCCCGCGTCTGATTAGGTAGTTGGTAGGGTAACGGCCTACCAAGCCGACGATCAGTAGCCGACCTGAGAGGGTGACCGGCCACATTGGGACTGAGACACGGCCCAAACTCCTACGGGAGGCAG

>ref23

GATGAACGCTGGCGGCGTGCTTAACACATGCAAGTCGAACGAAGCACTTAAGTTTGATTCTTCGGATGAAGACTTTTGTGACTGAGTGGCGGACGGGTGAGTAACGCGTGGGTAACCTGCCTCATACAGGGGGATAACAGTTAGAAATGACTGCTAATACCGCATAAGACCACAGCACCGCATGGTGCAGGGGTAAAAACTCCGGTGGTATGAGATGGACCCGCGTCTGATTAGCTGGTTGGTGGGGTAACGGCCTACCAAGCGACGATCAGTAGCCGACCTGAGAGGGTGACCGGCCACATTGGGACTGAGACACGGCCCAAACTCCTACGGGAGGCAG

>ref24

GATGAACGCTGGCGGCGTGCTTAACACATGCAAGTCGAACGAAGCACTTAAGTTTGATTCTTCGGATGAAGACTTTTGTGACTGAGTGGCGGACGGGTGAGTAACGCGTGGGTAACCTGCCTCATACAGGGGGATAACAGTTAGAAATGGCTGCTAATACCGCATAAGACCACAGCACCGCATGGTGCAGGGGTAAAAACTCCGGTGGTATGAGATGGACCCGCGTCTGATTAGCTAGTTGGTGGGGTAACGGCCTACCAAGGCGACGATCAGTAGCCGACCTGAGAGGGTGACCGGCCACATTGGGACTGAGACACGGCCCAAACTCCTACGGGAGGCAG

>ref25

GATGAACGCTGGCGGCGTGCTTAACACATGCAAGTCGAACGAAGCACTTAAGTTTGATTCTTCGGATGAAGACTTTTGTGACTGAGTGGCGGACGGGTGAGTAACGCGTGGGTAACCTGCCTCATACAGGGGGATAACAGTTAGAAATGGCTGCTAATACCGCATAAGACCACAGCACCGCATGGTGCAGGGGTAAAAACTCCGGTGGTATGAGATGGACCCGCGTCTGATTAGCTGGTTGGTGGGGTAACGGCCTACCAAGGCGACGATCAGTAGCCGACCTGAGAGGGTGACCGGCCACATTGGGACTGAGACACGGCCCAAACTCCTACGGGAGGCAG

>ref26

GATGAACGCTGGCGGCGTGCTTAACACATGCAAGTCGAACGAAGCAGTACTGTGTGAAGAGATTAGCTTGCTAAGATCAGAACTTTGTATTGACTGAGTGGCGGACGGGTGAGTAACGCGTGGGCAACCTGCCTTACACAGGGGGATAACAGCTAGAAATGGCTGCTAATACCGCATAAGACCTCAGTACCGCATGGTAGAGGGGTAAAAACTCCGGTGGTGTAAGATGGGCCCGCGTCTGATTAGGTAGTTGGTAGGGTAACGGCCTACCAAGCCGACGATCAGTAGCCGACCTGAGAGGGTGACCGGCCACATTGGGACTGAGACACGGCCCAAACTCCTACGGGAGGCAG

>ref27

GATGAACGCTGGCGGCGTGCTTAACACATGCAAGTCGAACGGAGCACCCCTGACGGAGTTTTCGGACAACGAAAGGGAATGCTTAGTGGCGGACGGGTGAGTAACGCGTGAGTAACCTGCCTTGGAGTGGGGAATAACAGCCGGAAACGGCTGCTAATACCGCATGATGTATCTGGATCGCATGGTTCTGGATACCAAAGATTTATCGCTCTGAGATGGACTCGCGTCTGATTAGCTAGTTGGTGAGGTAACGGCTCACCAAGGCGACGATCAGTAGCCGGACTGAGAGGTTGGCCGGCCACATTGGGACTGAGACACGGCCCAGACTCCTACGGGAGGCAG

>ref28

GATGAACGCTGGCGGCGTGCTTAACACATGCAAGTCGAGCGAAGCGCCTTATACAGATTCCTTCGGGATGAAGGATATGGCGACTGAGCGGCGGACGGGTGAGTAACGCGTGGGCAACCTGCCTCACACAGGGGGATAACAGCTAGAAATGGCTGCTAATACCGCATAAGGCCGCGGCACCGCATGGTGCGGCGGCAGAAGCTCCGGCGGTGTGAGATGGGCCCGCGTCTGATTAGGTAGTTGGTGCGGTAACGGCGCACCAAGCCGACGATCAGTAGCCGACCTGAGAGGGTGACCGGCCACATTGGGACTGAGACACGGCCCAAACTCCTACGGGAGGCAG

>ref29

GATGAACGCTGGCGGCGTGCTTAACACATGCAAGTCGAGCGAAGCGCCTTATACAGATTCCTTCGGGATGAAGGATTTGGCGACTGAGCGGCGGACGGGTGAGTAACGCGTGGGCAACCTGCCTCACACAGGGGGATAACAGCTAGAAATGGCTGCTAATACCGCATAAGGCCGCGGCACCGCATGGTGCGGCGGCAGAAGCTCCGGCGGTGTGAGATGGGCCCGCGTCTGATTAGGTAGTTGGTGCGGTAACGGCGCACCAAGCCGACGATCAGTAGCCGACCTGAGAGGGTGACCGGCCACATTGGGACTGAGACACGGCCCAAACTCCTACGGGAGGCAG
