# Supplementary material for: Spatial distribution of live gut microbiota and bile acid metabolism in various parts of human large intestine
Source: Sci Rep. 2022 Mar 4;12:3593. doi: 10.1038/s41598-022-07594-6 (PMC8897406; doi:10.1038/s41598-022-07594-6)
Supplement: Supplementary file 2 — Supplementary Information 2. [file 41598_2022_7594_MOESM2_ESM.docx]

**Supplementary materials**

**Spatial distribution of live gut microbiota and bile acid metabolism in various parts of human large intestine**

Daisuke Chinda^a, b^*, Toshihiko Takada^c^, Tatsuya Mikami^d^, Kensuke Shimizu^c^, Kosuke Oana^c^, Tetsu Arai^a^, Kazuki Akitaya^a^, Hirotake Sakuraba^a^, Miyuki Katto^c^, Yusuke Nagara^c^, Hiroshi Makino^e^, Daichi Fujii^f^, Kenji Oishi^c^, and Shinsaku Fukuda^a^

*^a^Department of Gastroenterology and Hematology, Hirosaki University Graduate School of Medicine, Aomori, Japan; ^b^Division of Endoscopy, Hirosaki University Hospital, Aomori, Japan; ^c^Basic Research Department, Yakult Central Institute, Tokyo, Japan; ^d^Innovation Center for Health Promotion, Hirosaki University Graduate School of Medicine, Aomori, Japan; ^e^Food Research Department, Yakult Central Institute, Tokyo, Japan; ^f^Microbiological Research Department, Yakult Central Institute, Tokyo, Japan*

*Corresponding Author: Daisuke Chinda

Department of Gastroenterology and Hematology, Hirosaki University Graduate School of Medicine, 5 Zaifu-cho, Hirosaki 036-8562, Japan

Tel: +81-172-39-5053; Fax: +81-172-37-5946; e-mail: [chinda@hirosaki-u.ac.jp](mailto:chinda@hirosaki-u.ac.jp)

**Supplementary table 1**

Taxonomic annotation of the features assumed to be *bai*-exhibiting

| **Taxonomic annotation** | **Similarity (%)** |
| --- | --- |
| *[Clostridium] scindens* | 100.0 |
| *[Clostridium] scindens* | 99.7 |
| *[Clostridium] scindens* | 95.1 |
| *[Clostridium] scindens* | 95.1 |

S**upplementary table 2**

Reference sequences of *bai*-exhibiting/non-*bai*-exhibiting bacteria

| **Source** | **Name** | **ID** | ***bai*** | **seq_ID** |
| --- | --- | --- | --- | --- |
| NCBI | Clostridiales bacterium UBA1701 | GCA_002320035.1 | ＋ | ND |
|  | Clostridiales bacterium UBA4701 | GCA_002404685.1 | ＋ | ND |
|  | Clostridiales bacterium UBA6412 | GCA_002439785.1 | ＋ | ND |
|  | Clostridiales bacterium UBA7103 | GCA_002493305.1 | ＋ | ND |
|  | Clostridiales bacterium VE202-26 | GCA_000509125.1 | ＋ | ND |
|  | Clostridiales bacterium_UBA5888 | GCA_002431965.1 | ＋ | ND |
|  | *Clostridium hiranonis* DSM 13275 | GCF_000156055.1 | ＋ | ND |
|  | *Clostridium hylemonae* DSM 15053 | GCF_008281175.1 | ＋ | ref22, ref26 |
|  | *Clostridium scindens* ATCC 35704 | GCF_004295125.1 | ＋ | ref05, ref21 |
|  | *Clostridium scindens* VE202-05 | GCA_000471845.1 | ＋ | ND |
|  | *Clostridium sordellii* VPI 9048 | GCF_000444095.1 | ＋ | ND |
|  | *Clostridium* sp. AF15-17LB | GCF_003464355.1 | ＋ | ref16 |
|  | *Clostridium* sp. Marseille-P2538 | GCF_900086625.1 | ＋ | ref28, ref29 |
|  | *Clostridium* sp. strain 2789STDY5834951 | GCA_900066925.1 | ＋ | ref09 |
|  | *Dorea formicigenerans* AF12-11 | GCF_003465045.1 | − | ref15 |
|  | *Dorea longicatena* AF17-8AC | GCF_003460005.1 | − | ND |
|  | *Dorea* sp. AF36-15AT | GCF_003477705.1 | − | ref12 |
|  | *Dorea* sp. AM58-8 | GCF_003464045.1 | ＋ | ref23 |
|  | *Dorea* sp. D27 | GCF_001185345.1 | ＋ | ref17 |
|  | *Eubacterium* sp. strain 2789STDY5834872 | GCA_900066555.1 | ＋ | ref24 |
|  | Firmicutes bacterium CAG:103 | GCA_000432375.1 | ＋ | ND |
|  | Lachnospiraceae bacterium 5_1_57FAA | GCA_000218425.1 | ＋ | ref05 |
|  | *Paeniclostridium sordellii* AF05-25 | GCF_003465925.1 | − | ref06 |
|  | *Peptoclostridium* sp. AF21-18 | GCF_003478825.1 | ＋ | ref08 |
|  | *Proteocatella sphenisc*i DSM 23131 | GCF_000423525.1 | ＋ | ND |
|  | *Pseudoflavonifractor* sp. AF19-9AC | GCA_003478995.1 | − | ref14 |
|  | *Romboutsia* sp. MT17 | GCF_900074625.1 | ＋ | ND |
|  | Ruminococcaceae bacterium AM07-15 | GCA_003477405.1 | − | ref19 |
|  | Ruminococcaceae bacterium AM28-23LB | GCA_003480625.1 | − | ref18 |
|  | Ruminococcaceae bacterium TF06-43 | GCA_003481145.1 | − | ND |
|  | *Ruminococcus* sp. strain 2789STDY5608817 | GCA_900066095.1 | ＋ | ref25 |
| Pasolli et al. [1] | CosteaPI_2017__SID713A002-11-0-0__bin.25 | 15266 | ＋ | ND |
|  | CosteaPI_2017__SID713A023-11-0-0__bin.14 | 15260 | ＋ | ND |
|  | CosteaPI_2017__SID713B026-11-90-0__bin.59 | 15265 | ＋ | ND |
|  | CosteaPI_2017__SID713B072-11-0-0__bin.31 | 15194 | − | ND |
|  | FengQ_2015__SID31866__bin.46 | 15202 | ＋ | ref02 |
|  | FengQ_2015__SID31866__bin.65 | 15004 | ＋ | ND |
|  | FengQ_2015__SID530348__bin.18 | 6132 | − | ND |
|  | FengQ_2015__SID531416__bin.49 | 15210 | ＋ | ND |
|  | GeversD_2014__SKBSTL008__bin.71 | 15267 | ＋ | ND |
|  | KarlssonFH_2013__S54__bin.27 | 15188 | ＋ | ND |
|  | LiJ_2014__MH0358__bin.56 | 15005 | ＋ | ND |
|  | LomanNJ_2013__OBK1196__bin.13 | 4631 | ＋ | ND |
|  | LoombaR_2017__SID5395_kej__bin.39 | 4552 | ＋ | ND |
|  | LoombaR_2017__SID5639_uuc__bin.37 | 4622 | − | ND |
|  | Obregon-TitoAJ_2015__NO11__bin.28 | 4621 | ＋ | ND |
|  | Obregon-TitoAJ_2015__SM11__bin.2 | 15190 | ＋ | ND |
|  | QinJ_2012__CON-102__bin.13 | 6148 | − | ND |
|  | QinJ_2012__T2D-092__bin.53 | 4633 | ＋ | ND |
|  | QinN_2014__LD-84__bin.30 | 6131 | ＋ | ND |
|  | RaymondF_2016__P21E7__bin.22 | 15209 | ＋ | ref03 |
|  | RaymondF_2016__P2E7__bin.68 | 15177 | − | ND |
|  | RaymondF_2016__P6C0__bin.19 | 15195 | − | ND |
|  | SchirmerM_2016__G89065__bin.22 | 15264 | ＋ | ND |
|  | VogtmannE_2016__MMRS15137911ST-27-0-0__bin.3 | 15211 | ＋ | ND |
|  | XieH_2016__YSZC12003_35390__bin.5 | 15003 | ＋ | ND |
|  | XieH_2016__YSZC12003_36474__bin.34 | 15196 | − | ND |
|  | XieH_2016__YSZC12003_37181R1__bin.102 | 15244 | ＋ | ND |
|  | YuJ_2015__SZAXPI017595-169__bin.30 | 4630 | ＋ | ND |
| Almeida et al. [2] | ERR1018198_bin.24 | ERZ852679 | ＋ | ND |
|  | ERR1018212_bin.42 | ERZ860007 | ＋ | ND |
|  | ERR1190589_bin.37 | ERZ878070 | ＋ | ND |
|  | ERR1190593_bin.31 | ERZ878148 | ＋ | ND |
|  | ERR1190714_bin.6 | ERZ879681 | − | ND |
|  | ERR1297716_bin.14 | ERZ874464 | ＋ | ND |
|  | ERR1305880_bin.25 | ERZ833022 | ＋ | ND |
|  | ERR1305892_bin.4 | ERZ833433 | ＋ | ND |
|  | ERR1305903_bin.25 | ERZ833919 | ＋ | ND |
|  | ERR1620278_bin.11 | ERZ849836 | − | ND |
|  | ERR1620300_bin.15 | ERZ850250 | ＋ | ND |
|  | ERR1913015_bin.15 | ERZ867858 | ＋ | ref01 |
|  | ERR414305_bin.35 | ERZ896748 | ＋ | ND |
|  | ERR526064_bin.3 | ERZ904696 | ＋ | ND |
|  | ERR589534_bin.49 | ERZ876179 | ＋ | ND |
|  | ERR688523_bin.66 | ERZ865653 | ＋ | ND |
|  | SRR1761702_bin.21 | ERZ837753 | ＋ | ND |
|  | SRR2155369_bin.7 | ERZ844558 | ＋ | ND |
|  | SRR2155387_bin.7 | ERZ844771 | − | ND |
|  | SRR2726136_bin.31 | ERZ832586 | ＋ | ND |
|  | SRR2912788_bin.43 | ERZ834790 | ＋ | ref10 |
|  | SRR2912789_bin.15 | ERZ834824 | ＋ | ND |
|  | SRR2912810_bin.38 | ERZ835316 | ＋ | ND |
|  | SRR2992893_bin.21 | ERZ862544 | ＋ | ref20 |
|  | SRR3737028_bin.26 | ERZ838947 | ＋ | ND |
|  | SRR5057034_bin.63 | ERZ945601 | ＋ | ND |
|  | SRR5077148_bin.27 | ERZ946943 | ＋ | ND |
|  | SRR5091468_bin.55 | ERZ866808 | ＋ | ND |
|  | SRR5275456_bin.18 | ERZ843436 | ＋ | ND |
|  | SRR6028174_bin.3 | ERZ979246 | ＋ | ND |
| Forster et al. [3] | *Intestinimonas butyriciproducens* ERR1022450 | ERR1022450 | − | ref27 |
|  | *Lachnoclostridium* nov. ERR1022276 | ERR1022276 | ＋ | ND |
|  | *Lachnoclostridium* nov. ERR1022388 | ERR1022388 | ＋ | ND |
|  | *Lachnoclostridium* nov. ERR1203939 | ERR1203939 | ＋ | ND |
|  | *Lachnoclostridium* nov. ERR1204033 | ERR1204033 | ＋ | ND |
|  | *Lachnoclostridium* nov. ERR171273 | ERR171273 | ＋ | ref21 |
|  | *Lachnoclostridium* nov. ERR2230109 | ERR2230109 | ＋ | ND |
|  | *Lachnoclostridium* nov.ERR1022434 | ERR1022434 | − | ref13 |
|  | *lntestinibacter bartlettii* ERR1022418 | ERR1022418 | − | ND |
|  | *Oscillibacter* nov. ERR1022407 | ERR1022407 | − | ref04 |
|  | *Peptoclostridium difficile* ERR2221219 | ERR2221219 | − | ref07 |
|  | *Peptoclostridium difficile* ERR2221225 | ERR2221225 | − | ref11 |
|  | *Pseudoflavonifractor capillosus* ERR1022447 | ERR1022447 | − | ND |
|  | *Romboutsia* nov. ERR1022375 | ERR1022375 | − | ND |
|  | *Romboutsia* nov. ERR1022465 | ERR1022465 | ＋ | ND |
|  | *Romboutsia* nov. ERR1022466 | ERR1022466 | ＋ | ref09 |
|  | *Terrisporobacter mayombei* ERR2221147 | ERR2221147 | − | ND |

name; genome names as reported by Vital et al. (2019). [4]

ID; accession number, SGB id, ENA Run accession, ENA Analysis accession

bai; “+” means *bai*-exhibiting and “−” means non-*bai*-exhibiting according to Vital et al. [4]

Seq_ID; name of the V1-V2 regions of the 16S rRNA gene sequence in the supplementary fasta file

1. Pasolli, E. *et al.* Extensive unexplored human microbiome diversity revealed by over 150,000 genomes from metagenomes spanning age, geography, and lifestyle. *Cell*. **176**, 649-662 (2019).

2. Almeida, A. *et al*. A new genomic blueprint of the human gut microbiota. *Nature*. 499-504 (2019).

3. Forster, SC. *et al*. A human gut bacterial genome and culture collection for improved metagenomic analyses. *Nat Biotechnol*. **37**, 186-192 (2019).

4. Vital, M., Rud, T., Rath, S., Pieper, D.H. & Schlüter, D. Diversity of bacteria exhibiting bile acid-inducible 7α-dehydroxylation genes in the human gut. Comput. Struct. Biotechnol. J. 17, 1016-1019 (2019).


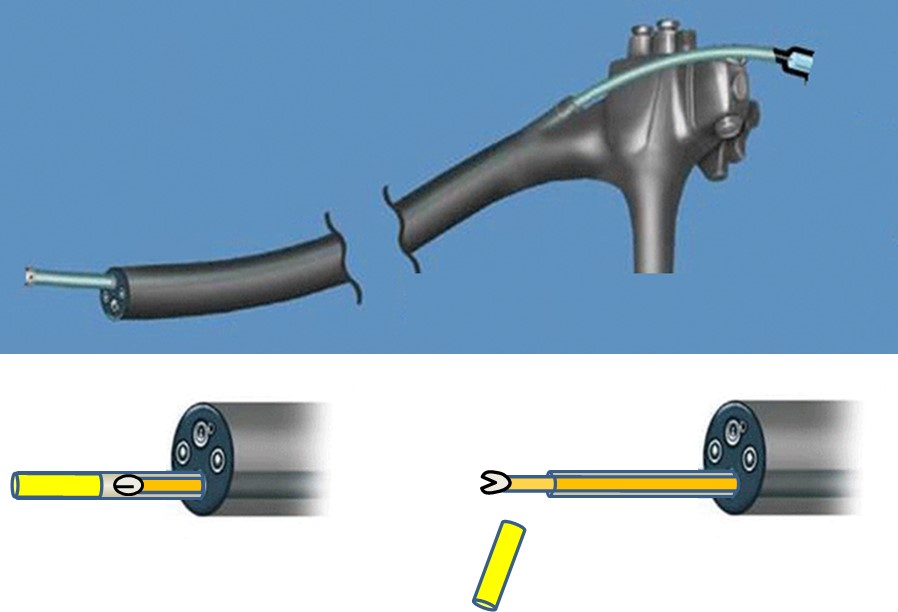


**Supplementary Figure 1.** Tool used to sample the luminal and mucosal contents from each part of the gut. The diagram shows a schematic of the biopsy forceps used to sample the gut contents. A scrape cytology brush for endoscopy was used to obtain mucosal scraping samples. The tip of the outer cylinder of the sampling instrument was sealed with beeswax to prevent contamination and pushed out during sampling.


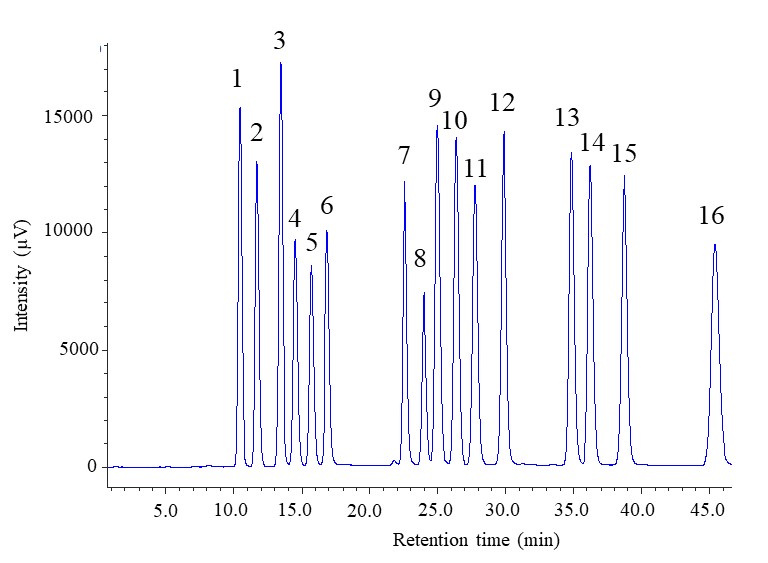


**Supplementary Figure 2.** HPLC chromatographic patterns of the standard bile acid. The number on each peak represents the compound number in the appendix: 1. Glycoursodeoxycholic acid (GUDCA), 2. Tauroursodeoxycholic acid (TUDCA), 3. Ursodeoxycholic acid (UDCA), 4. Glycocholic acid (GCA), 5. Taurocholic acid (TCA), 6. Cholic acid (CA), 7. Glycochenodeoxycholic acid (GCDCA), 8. Taurochenodeoxycholic acid (TCDCA), 9. Glycodeoxycholic acid (GDCA), 10. Taurodeoxycholic acid (TDCA), 11. Chenodeoxycholic acid (CDCA), 12. Deoxycholic acid (DCA), 13. Glycolithocholic acid (GLCA), 14. Taurolithocholic acid (TLCA), 15. 5β-pregnan-3α,17α,20α-triol (Internal standard), 16. Lithocholic acid (LCA)

Figure 3
